# Phylogeography of Chinese cereal cyst nematodes sheds lights on their origin and dispersal

**DOI:** 10.1111/eva.13452

**Published:** 2022-08-04

**Authors:** Xue Qing, Huan Peng, Jukui Ma, Yuanmeng M. Zhang, Hongmei Li, Deliang Peng, Xuan Wang, Tengwen Long

**Affiliations:** ^1^ Department of Plant Pathology Nanjing Agricultural University Nanjing China; ^2^ Key Laboratory of Integrated Management of Crop Disease and Pests, Ministry of Education Nanjing Agricultural University Nanjing China; ^3^ Institute of Plant Protection Chinese Academy of Agricultural Sciences Beijing China; ^4^ Key Laboratory of Biology and Genetic Improvement of Sweet Potato, Ministry of Agriculture, Xuzhou Institute of Agricultural Sciences in Jiangsu Xuhuai Area Jiangsu Xuzhou Sweet Potato Research Center Xuzhou Jiangsu China; ^5^ Systematic Entomology Laboratory USDA‐ARS, c/o National Museum of Natural History Washington District of Columbia USA; ^6^ School of Geographical Sciences University of Nottingham Ningbo China Ningbo China

**Keywords:** cereal cyst nematodes, *Heterodera avenae*, *Heterodera pratensis*, molecular species delimitation, phylogeny, population structure

## Abstract

Reconstructing the dispersal routes of pathogens can help identify the key drivers of their evolution and provides a basis for disease control. The cereal cyst nematode *Heterodera avenae* is one of the major nematode pests on cereals that can cause 10%–90% crop yield losses worldwide. Through extensive sampling on wheat and grasses, the Chinese population of *H. avenae* is widely identified in virtually all wheat growing regions in China, with H1 being the predominant haplotype. The monoculture of wheat in north China might have been the key driver for the prevalence of H1 population, which should date no earlier than the Han Dynasty (202 BCE–220 CE). Molecular phylogenetic and biogeographic analyses of Chinese *H. avenae* suggest a Pleistocene northwest China origin and an ancestral host of grasses. We assume that the prosperity of *Heterodera* in this region is a result of their preference for cooler climate and various grass hosts, which only appeared after the uplift of Qinghai‐Tibetan Plateau and aridification of Inner Asia. Nematode samples from the current and historical floodplains show a significant role of the Yellow River in the distribution of Chinese *H. avenae*. Whereas mechanical harvesters that operate on an inter‐provincial basis suggest the importance in the transmission of this species in eastern China in recent times. This study highlights the role of environmental change, river dynamics, and anthropogenic factors in the origin and long‐distance dissemination of pathogens.

## 1 INTRODUCTION

For thousands of years, small grain cereals such as wheat, barley, and oats have served as the basis of staple foods, beverages, and animal feed. Supplying calories consumed by people worldwide, as a primary source of energy for humans, played a vital role in global food and nutrition security (Smiley et al., 2017). The production of cereals is limited by various biotic and abiotic constraints, with plant‐parasitic nematodes alone being estimated to reduce production of all world crops by 10% (Whitehead, 1998). As one of the major nematode pests on cereal crops including wheat, barley, and oats, the cereal cyst nematodes *Heterodera avenae* Wollenweber, 1924 (Order: Rhabditida, Family: Hoplolaimidae) has been reported from nearly 40 countries (Balwin & Mundo‐Ocampo, 1991; Rivoal & Cook, 1993), infecting more than 50% of the European cereal growing areas and 22% of the Chinese wheat fields (Peng et al., 2009; Rivoal & Cook, 1993), with losses to yield ranging from 10% to 90% (Dababat et al., 2015; Peng et al., 2009; Riley & McKay, 2009).

China is the world's largest producer of wheat, producing more than 136.9 Mt yield per year (National Bureau of Statistics, 2021). *Heterodera avenae* was first reported in China in 1989 (Chen et al., 1991) and is widely distributed in the major wheat‐producing regions of 16 provinces (Cui et al., 2015; Peng et al., 2009). These Chinese *H. avenae* populations (CHA) differ considerably from European *H. avenae* populations (EHA) in pathotypes (Peng & Cook, 1996; Zheng et al., 1997) and iso‐electric focusing protein profiles (Sturhan & Rumpenhorst, 1996). Subsequently, Subbotin et al. (2003, 2010) discovered that nematode samples identified as “*H. avenae*” from China appeared to form a distinct group within the *H. avenae* complex. These CHA were further distinguished morphologically and identified as genetically to *H. pratensis* Gäbler et al. (2000), and finally described as a new species *H. sturhani* Subbotin, (2015). Although the validation of *H. sturhani* as a separate species remains in doubt (Peng, Holgado, et al., 2016; Peng, Li, et al., 2016), it is clear that CHA populations represent a unique evolutionary lineage in comparison with European *H. avenae* populations (Subbotin et al., 2018). This gives rise to the question for the origin of CHA.

Compared with the wide distribution of EHA population, CHA was only found in China. Subbotin et al. (2018) showed that CHA has low mtCOI genetic diversity based on limited samples. This suggests that CHA may have gone through a strong bottleneck as a result of being associated with a domesticated host, and thus their dissemination may associate with its host. However, no further evidence supports this, and consequently, their dispersal pathway remains unclear.

In the present study we performed a country‐wide sampling across nearly 10 years, covering all major wheat growing areas as well as several grasslands in China. Mitochondrial cytochrome c oxidase subunit 1 (mtCOI) gene was sequenced to reconstruct population structure, and the microsatellites in expressed sequence tags (ESTs) were further examined to verify the recovered pattern. Through phylogeographic and population genetic analyses, we aim to elucidate the following: (i) the clear genetic structure of CHA across China, (ii) the geographical and ecological origin of CHA, and (iii) its primary dispersal pathway.

## 2 MATERIALS AND METHODS

### 2.1 Sampling and nematode extraction

A total of 633 sites were sampled from wheat field and grass during several surveys between 2010 and 2021, which covered nearly all major wheat growing areas of China as well as alpine meadow in northwest China (Tables S1–S4). Since the major CHA distribution regions overlap the Yellow River Basin of China, the roles of the Yellow River in CHA dissemination were suspected. Subsequently, we sampled 16 sites along the upper, middle, and lower reaches of the Yellow River, as well as its tributaries and irrigation ditches. We considered individual cysts from one site as the same population, and subsequently, these recovered population assigned to 14 (mtCOI) or 13 (microsatellites markers, grass sample not included) regions according their geographic origin. Additionally, we also examined soil remnants on reels and wheels of mechanical harvesters crossing regional and provincial for the presence of cysts (Figures S1–S4), and the samples were mainly collected from 47 different locations (Table S2) in Henan and Hebei provinces where machine harvesting is widely utilized. The methodology related to morphological analyses is provided in Appendix S4.

### 2.2 DNA extraction, PCR amplification, and sequencing

DNA was extracted from the eggs and second‐stage juveniles (J2s) released from a single cyst. Cysts were soaked in double distilled water for 10–20 min in advance, and one cyst was put into 20 μl ddH_2_O on a sterilized glass slide, punctured by a needle under a dissecting microscope. The released eggs and J2s were crushed by sterilized needle and transferred together with water suspension into an Eppendorf tube containing 20 μl of worm lysis buffer solution (50 mM KCl; 10 mM Tris pH 8.3; 2.5 mM MgCl_2_; 0.45% NP 40; 0.45% Tween 20). The tube was frozen for at least 10 min at −20°C, and 1 μl proteinase K (1.2 mg/ml) was added before incubation for 1 h at 65°C and 10 min at 95°C. After incubation, the tube was centrifuged and kept at −20°C until use. A fragment of the mtCOI gene was amplified using two primer pairs, the details are listed in Appendix S4. To complement the mtCOI‐based population genetic study, for EST‐derived SSR microsatellite analysis, the markers and the protocol from Wang et al. (2018) were used. The details for this method are provided in Appendix S3.

### 2.3 Population structure and species delimitation

For mtCOI‐based study, the newly obtained sequences of CHA were analyzed together with other related sequences in downloaded from GenBank. Multiple alignments of mtCOI gene were generated using MAFFT implemented in TranslatorX (Abascal et al., 2010). The overhanging parts of long fragments (amplified with primer pairs COI442F/COI1326R) were truncated to the size of shortest mtCOI fragments (with JB3/JB4.5). The mtCOI haplotype diversity and population genetic structure were estimated using the PopART (Leigh & Bryant, 2015) by means of the minimum spanning networks.

Both long and short mtCOI fragments were used for phylogeny analyses, and duplicated haplotypes sequences were pruned from the dataset prior to analysis. The Bayesian inference analyses were performed in MrBayes 3.2.3 (Ronquist et al., 2012) using the GTR + I + G model. The GMYC species‐delimitation method (Pons et al., 2006) was performed in the R package Splits (Ezard et al., 2009) with a single threshold. An ultrametric tree was constructed using BEAST 2.6.2 (Bouckaert et al., 2019). The final output was examined in Tracer 1.7 (Rambaut et al., 2018), and ESS values more than 200 were considered as convergence. The output MCC tree was generated by TreeAnnotator 1.8 (Drummond et al., 2012) after removing 20% burn‐ins. ABGD was performed online (Puillandre et al., 2012), with the default program settings, and distances were calculated using the JC69 substitution model. The bPTP method (Zhang et al., 2013) was preformed online also using default settings (https://species.h‐its.org/).

For microsatellites‐based study, fixation index (*F*_ST_), the individual inbreeding coefficient (*F*_is_), as well as Analysis of Molecular Variance (AMOVA), were calculated among and within the populations. PCoA and Bayesian clustering were used to examine the population grouping. The possible bottleneck effect was tested based on three different models (IAM, TPM, and SMM). Population structure was inferred by using Bayesian clustering program STRUCTURE v2.3.4 (Falush et al., 2007; Pritchard et al., 2000). The CLUMPP v1.1.2 software (Jakobsson & Rosenberg, 2007) was used to calculate the Δ*K* (Evanno et al., 2005) and finally plotted using DISTRUCT (Rosenberg, 2004). More details on these methods were given in Appendix S3.

### 2.4 Divergence dating and historical biogeographic reconstruction

To understand the evolutionary history of CHA, divergence times were inferred with BEAST 2.6.2. Due to the lack of fossil calibration points, the molecular dating method was used following Subbotin et al. (2018). In brief, the tree prior to a lognormal relaxed clock with uncorrelated rates was assigned to the Yule model with the mitochondrial substitution genome rate equal to 7.2 × 10^−8^ per site per generation as calculated by Howe et al. (2010) for *Caenorhabditis briggsae* Dougherty and Nigon, 1949, and the life cycle with one generation per year. The analyses were run for 1 × 10^10^ generations and sampled every 10,000 generations. Convergence was monitored with Tracer 1.7.1 (Rambaut et al., 2018).

The possible ancestral ranges of CHA were reconstructed using S‐DIVA implemented in RASP 4.0 (Yu et al., 2020). The areas of occurrence were set as seven geographical regions: (A) central China, (B) northwest China, (C) western Europe, (D) USA, (E) Russia and Korea, (F) Australia and New Zealand, and (G) Middle East and Mediterranean. DEC + J was selected as the best model using weighted AICc in BioGeoBEARS, other tested models included DEC, DIVALIKE, BAYAREALIKE, and their +J counterparts (Matzke, 2014).

## 3 RESULTS

### 3.1 Distribution and population structure

The extensive investigation across China revealed that CHA is widely distributed in almost all wheat growing areas from 14 provinces, with 373 sites were positive (Tables S1–S4). Among them, mtCOI gene was successfully sequenced from 362 sites, while microsatellites analysis was performed based on 416 individual cysts (one to four cysts per site). Cysts were also found in 26 out of 47 soil samples (Table S2) attached to harvester reels and wheels, which indicated the machinery maybe an important tool for CHA transmission in Henan and Hebei provinces. Apart from the soil, we also examined the presence of cysts in water flow. Among 16 examined sites, 15 were positive, suggesting that cysts are rather common in the Yellow River, as well as its tributaries and irrigation ditches (Figure 2a,e).

**FIGURE 1:**
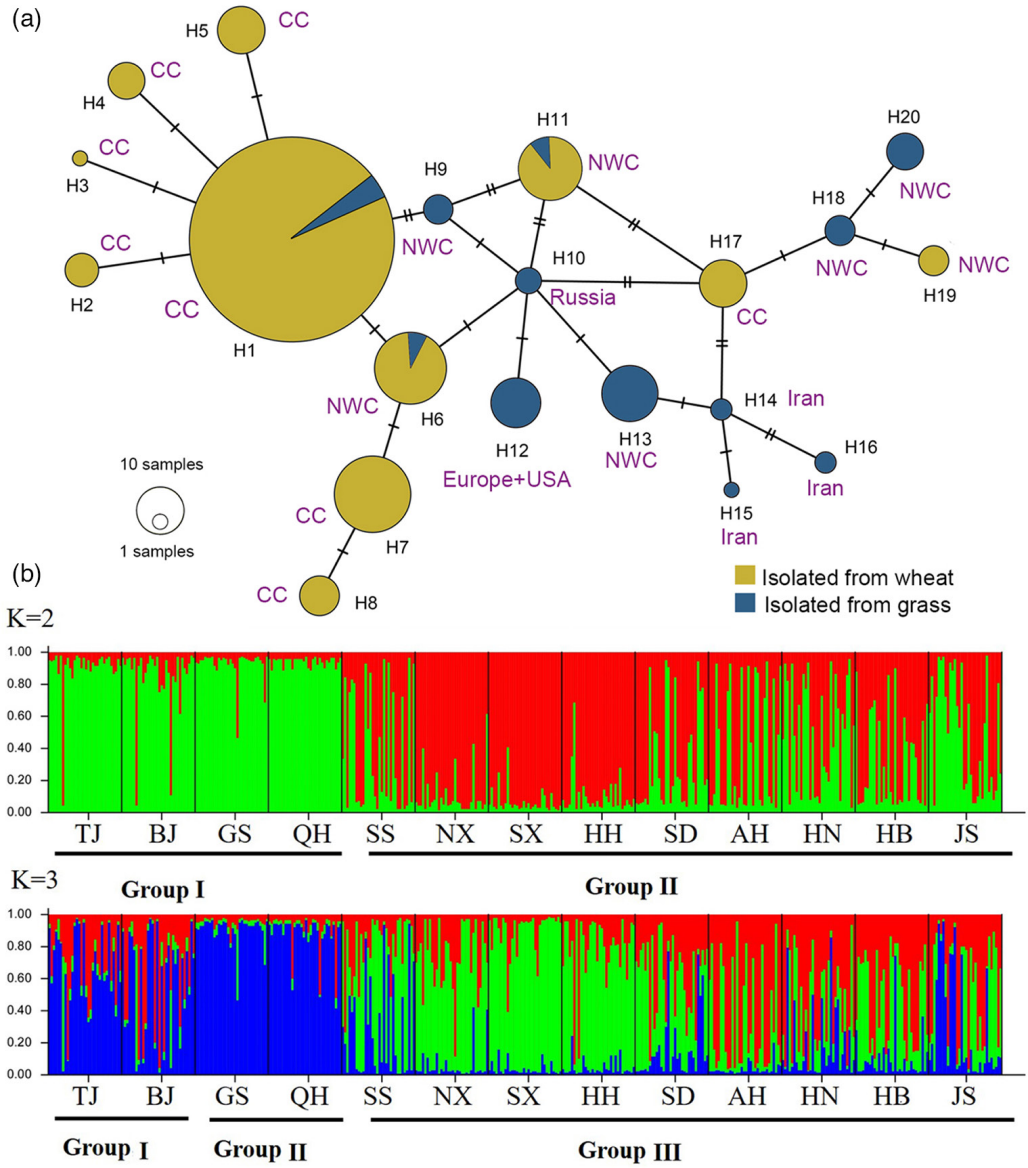
Population structure of cereal cyst nematodes inferred from mtCOI gene. (a) Minimum spanning haplotype network. Each circle corresponds to one haplotype and its size is proportional to its frequency. Each line connecting the haplotypes refers to a mutational step. Different colors indicate different types of host (wheat in yellow or grasses in blue) for each haplotype. The annotation next to the circle donates the geographic origin. CC, Central China; NWC, Northwest China. (b) Bayesian clustering using STRUCTURE program. The studied *Heterodera* spp. populations can be divided into two or four groups measured by Δ*K* and maximum posterior probability method. Mixed ancestries are shown by differently colored sectors, corresponding to inferred genetic percentages of the corresponding clusters. The abbreviations stand for the regions where the studied populations were collected (see Table S1 for details).

The mtCOI gene was obtained with 445 and 793 bp in length amplified by the two primers pairs. The subsequent analyses recovered 20 mtCOI haplotypes in the CHA + *H. pratensis* clade (GenBank accessions: ON357397–ON357427) (Figure 1a, Table S3). Among them, 12 haplotypes were newly discovered. Fourteen out of 15 Chinese haplotypes were endemic, with 1–15 nucleotides differences (Table S4), and one was also found in Korea (H9). Additionally, we found the presence of multiple haplotypes with intermediary to CHA and the rest of the *H. pratensis* complex, thus rejected CHA and *H. pratensis* as two distinct species. We also found that haplotypes previously defined as *H. sturhani* are prevalent in wheat, while haplotypes more related to *H. pratensis* are primarily found in grass. Exceptions are haplotypes H17 from central China and H19 from northwest China, which were solely recovered from wheat but genetically more related to *H. pratensis*.

**FIGURE 2:**
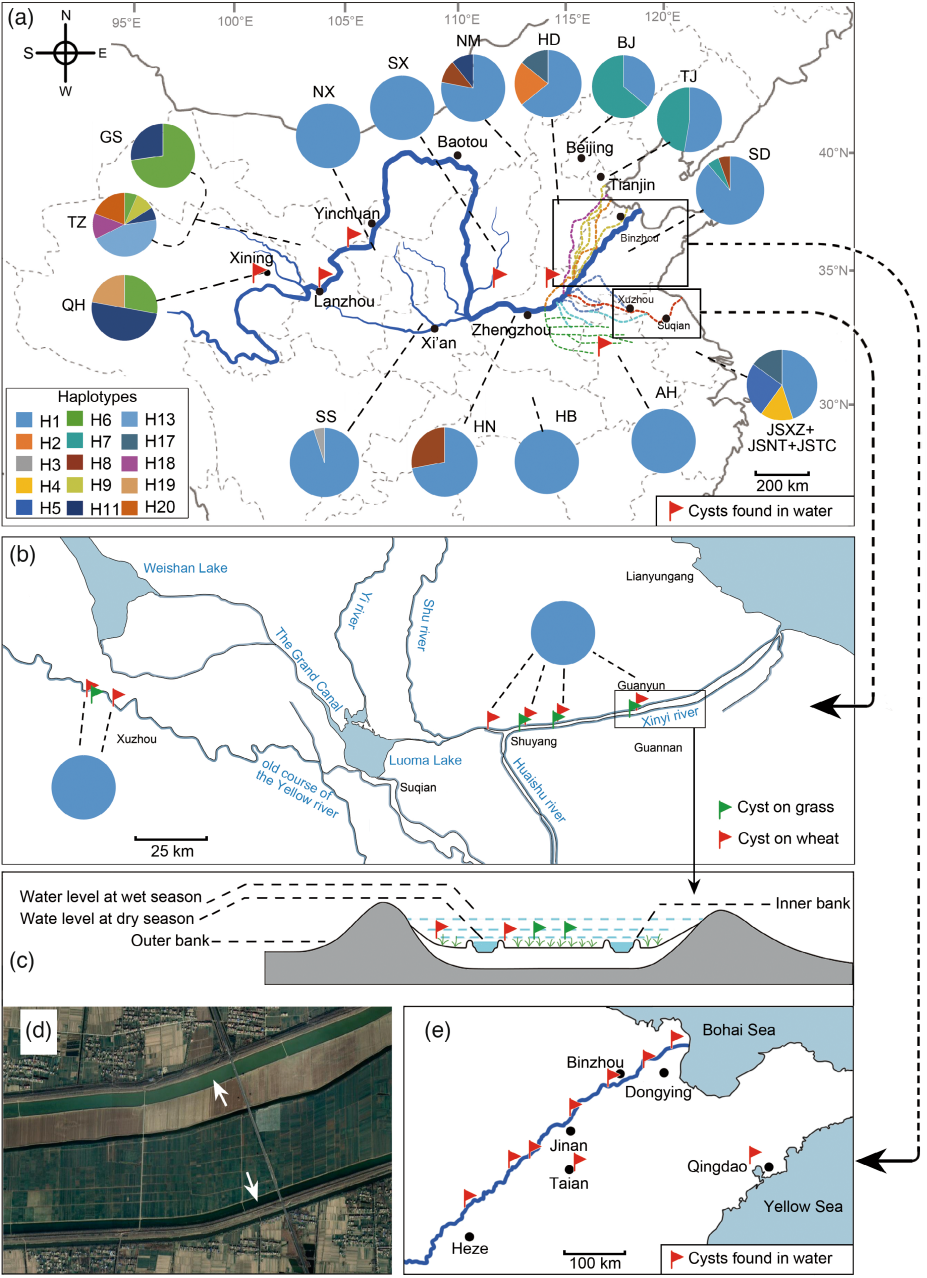
Haplotype compositions of Chinese cereal cyst nematode populations in different geographic regions of China. (a) Haplotype distribution in the Yellow River Basin, and the sites where cysts were caught from Yellow River, its tributaries, or irrigation ditches. The region abbreviations are given in Table S1. (b) Historical courses of the Yellow River and related river networks. The colors in the pie chart are proportional to the haplotype composition in each region. The colored dash line indicates historical courses of Yellow River, with details given in Figure 5k and c–e for Xinyi River in the Guanyun County, Jiangsu Province. (c) Cross section schematic of the river showing floodplain, seasonal rivers, and the locations where *Heterodera* spp. were recovered. In the dry season, wheat and wild grass grow on the floodplain between two inner rivers. In wet season, overbank flowing takes place when the inner river is flooded and the floodplain is fully submerged. (d) Google satellite view of the floodplain and seasonal rivers. Arrows point to two inner rivers. (e) Partial map of Shandong Province showing the sampling sites where cysts were observed in the water flow of the Yellow River or in irrigation ditches (two sites in Qingdao and Taian).

For recovered Chinese haplotypes, H1 was the most widespread haplotype that presented in all wheat growing regions except for Gangsu (GS), Qinghai (QH), and Tianzhu (TZ) population on grass (Figures 1a and 2a). The haplotype H1 was predominately parasitizing various wheat varieties, but in a rare case it was found on grass the *Roegneria kamoji* Ohwi next to wheat fields in the floodplain of Xinyi River of Jiangsu Province (Figure 2b–d). Likewise, haplotypes H6 and H11 were also found parasitizing both wheat and grass. Conversely, nine (H9, H10, H12–H16, H18, and H20) and eight (H2–H5, H7, H8, H17, H19) haplotypes were solely found either in grass or in wheat, respectively (Figure 1a), respectively. Regional analyses indicated that TZ contained the highest haplotype diversity (six haplotypes), while populations in Ningxia (NX), Shaanxi (SX), Hebei (HB), and Anhui (AH) all had only a single haplotype (H1).

The sampling of the old courses of the Yellow River suggested that CHA is widely distributed, with six independent populations being recovered (Figure 2b,c). These populations were found both on wheat and grass growing in the floodplain of the river as well as in fields outside the bank. The analysis of mtCOI gene showed all recovered populations sharing the same haplotypes H1, indicating a rapid population expansion may have occurred recently.

Using microsatellite markers, Bayesian clustering method suggested CHA can be split into two or three clusters (*K* = 2 or *K* = 3) without grass samples (Figure 1b). In two clustering, the populations from northwest China (GS and QH) lumped together with Beijing (BJ) and Tianjin (TJ) populations and distinct from other regions. Conversely, northwest China regions (GS and QH) were recognized as a unique group apart from BJ and TJ, and all other regions in three‐clustering model.

### 3.2 Phylogeny and species delimitation

The Bayesian phylogeny analysis suggested that newly recovered CHA populations were clustered together with *H. pratensis* in a moderate supported clade (PP = 80), sistering to *H. australis* (Figure 4a). Species delimitation was employed using GMYC, bPTP, and ABGD methods (Figure 4c). The number of recovered species varied by method, with 12 species using the single‐threshold GMYC model prediction, nine species based on bPTP, and between seven and ten species according to the different models and priors used in ABGD. In general, molecular species delimitations were largely congruent among themselves and with traditional methods, while few exceptions are remarkable. The sequences of CHA were grouped together with *H. pratensis* and recognized as a single species in all applied methods. The *H. australis* Subbotin, Sturhan, Rumpenhorst and Moens (2002), was nested inside the *H. pratensis* clade and considered a distinct species except for one of ABDG analyses (seven species grouping model). The European and Middle East populations of *H. avenae* were placed in a well‐supported clade (PP = 1), but appeared to be paraphyletic, with *H. arenaria* nested inside. The species delimitation analyses split this clade as five (GMYC), two (bPTP, ABDG), and one (ABGD) species. Although cryptic species may exist within *H. avenae*, none of them supporting *H. arenaria* as a valid species.

### 3.3 Divergence dating and historical biogeographic reconstruction

The divergence time and historical distribution estimates for each lineage are depicted in Figure 4b,d. According to our estimates, the last common ancestor of clade *H. pratensis* + CHA *+ H. australis* (node 1) was possibly a northwest China lineage that split from other cyst nematodes around 0.74–1.74 million years ago (mya) in the Pleistocene epoch. The clade consisting of major wheat‐parasitic CHA (node 2) diverged around 0.49–1.59 mya, probably from central China or northwest China. The lineage represented by typical grass‐parasitic populations (node 4) probably arose between 0.65 and 1.57 mya, while an intermediate clade contains both grass‐ and wheat‐parasitic populations (node 3) arose between 0.76 and 1.91 mya. Both nodes 3 and 4 may have a northwest China origin.

### 3.4 Morphology of different CHA populations

**FIGURE 3:**
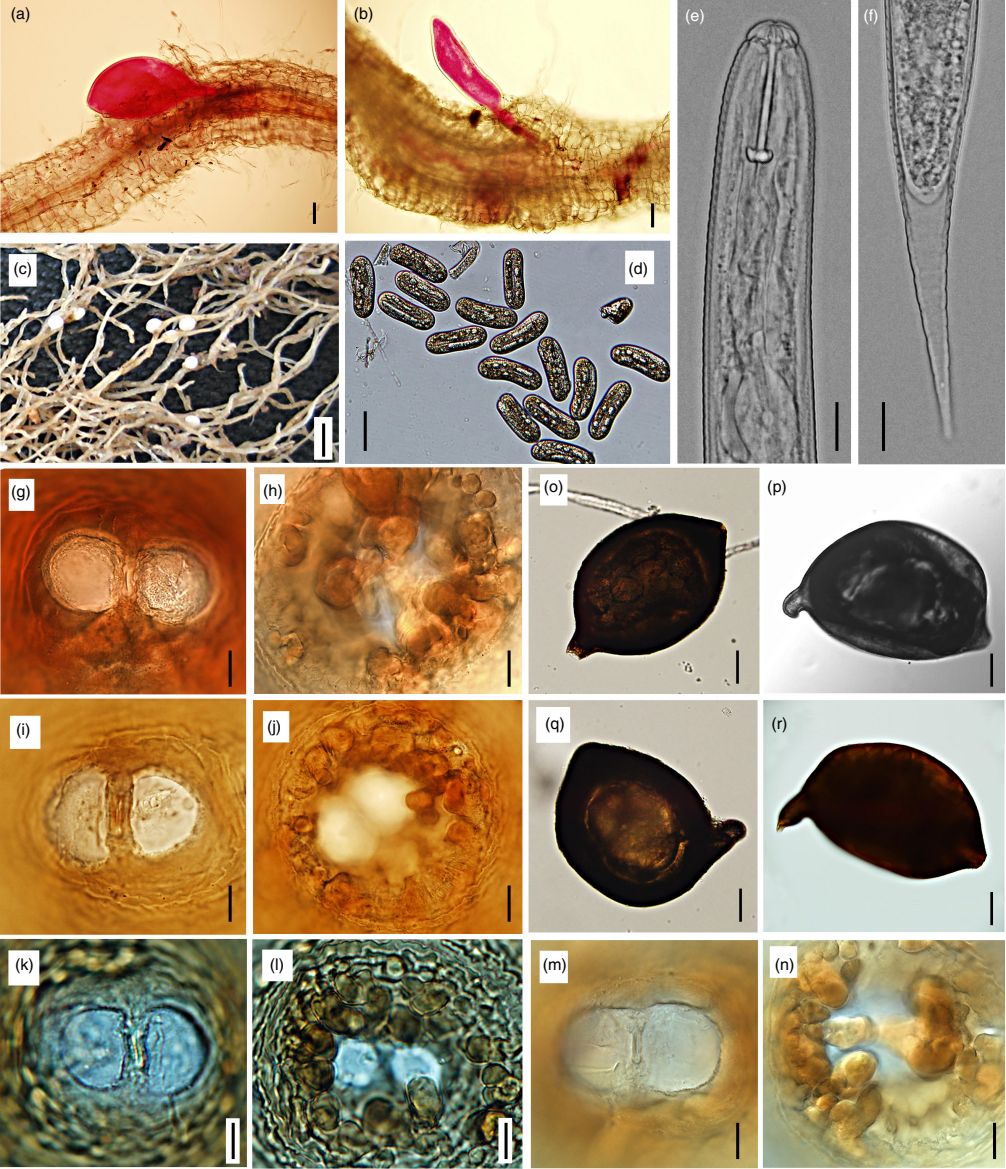
The infection process and general morphology of cereal cyst nematodes recovered in this study. (a) Fourth‐stage juvenile feeding from syncytium. (b) Third‐stage juvenile. (c) Swollen egg‐filled white female lodged in root tissue. (d) Eggs showing outlines of the J1 folded within the egg. (e) Head region of second‐stage juvenile. (f) Tail of second‐stage juvenile. (g–n) Fenestration (g, i, k, m) and underneath level view (h, g, l, n) of vulval cone for haplotypes H5 (g, h), H20 (i, j), H1 (k, l) and H13 (m, n); (o–r): Cysts extracted from soil for haplotypes H5 (o), H20 (p), H1 (q), H13 (r). Scale bar: A, b, o–r = 100 μm, c = 1 mm, d = 50 μm, e–l = 10 μm.

To assess morphological variations among different isolates and haplotypes, the cysts (Table S5) and second‐stage juveniles (J2, Table S6) were examined. In general, all recovered cysts and J2 fit the typical morphology of *H. avenae* without significant inter‐population variation (Figure 3). The cyst is lemon‐shaped, subcrystalline layer distinct, vulval cone bifenestrate, vulval slit short, bullae numerous, distinct. The J2 is slightly ventrally curved, labial region flatly rounded with two indistinct annuli, labial framework strongly sclerotized, and lateral field having four lines. The morphometrical analysis revealed that all recovered cysts were smaller than the other populations of *H. avenae* (Subbotin et al., 2003), although they varied slightly among haplotypes (Table S5). The average cyst sizes of most widespread wheat parasite haplotype H1 ranged from 570 to 603 μm, similar to that from other reported CHA, while grass‐isolated population with H13 haplotype was larger with an average of 671 μm, similar to that of *H. pratensis* (Table S5). The populations with haplotypes H4 and H5 were genetically intimate to that with H1, and this was in line with their similar cysts morphometrics. Conversely, the populations with haplotypes H9 and H20 have smaller cysts, regardless of their exclusively isolating from grass and genetically more relating to *H. pratensis*.

**FIGURE 4:**
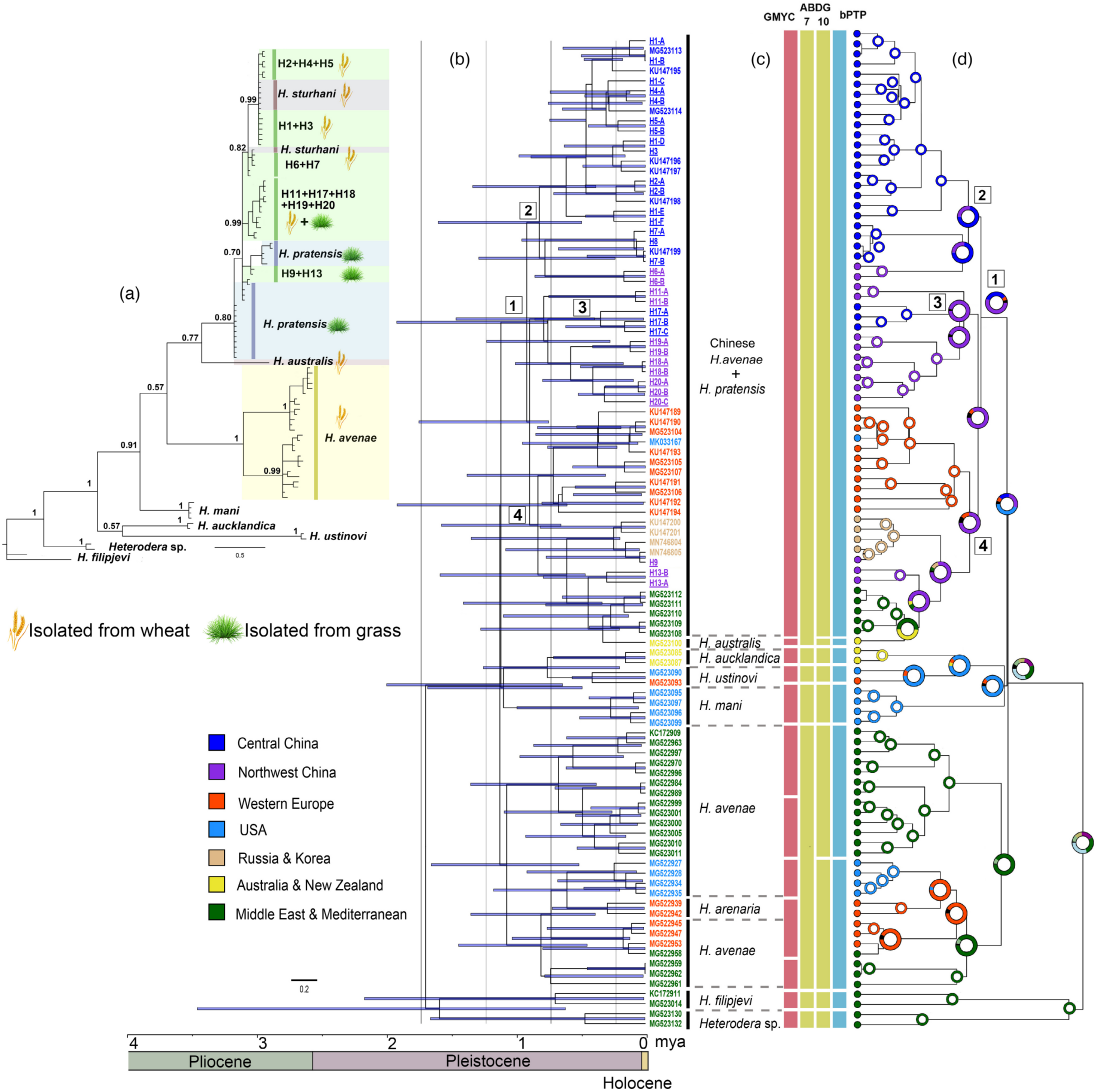
Phylogeny (a), divergence dating (b), molecular species‐delimitation (c) and historical biogeographic reconstruction analysis (d) of the cereal cyst nematodes using mtCOI gene marker. (a) mtCOI‐based phylogeny of *Heterodera* spp. using MrBayes. (b) Chronogram of *Heterodera* spp. based on BEAST analysis. Blue bars indicate 95% highest posterior density intervals. The terminal colors highlight individuals isolated from the same region. (c) Molecular species‐delimitation using three methods: GMYC, bPTP, and ABGD. For the ABGD analysis, groupings of 10 species and seven species are presented, as recovered based on different prior. (d) Reconstruction of the possible ancestral ranges of *Heterodera* species. The areas of occurrence were set as seven regions. The proportion of colors in a node circle is the probability of each region to be a historical distribution region. Nodes of interest are marked as (1): Chinese *H. avenae*, *H. pratensis* and *H. australis*, (2): The predominant CHA found in wheat, (3): The populations that parasitizing both grass and wheat, (4): The predominant grass parasitic populations including *H. pratensis*.

**FIGURE 5:**
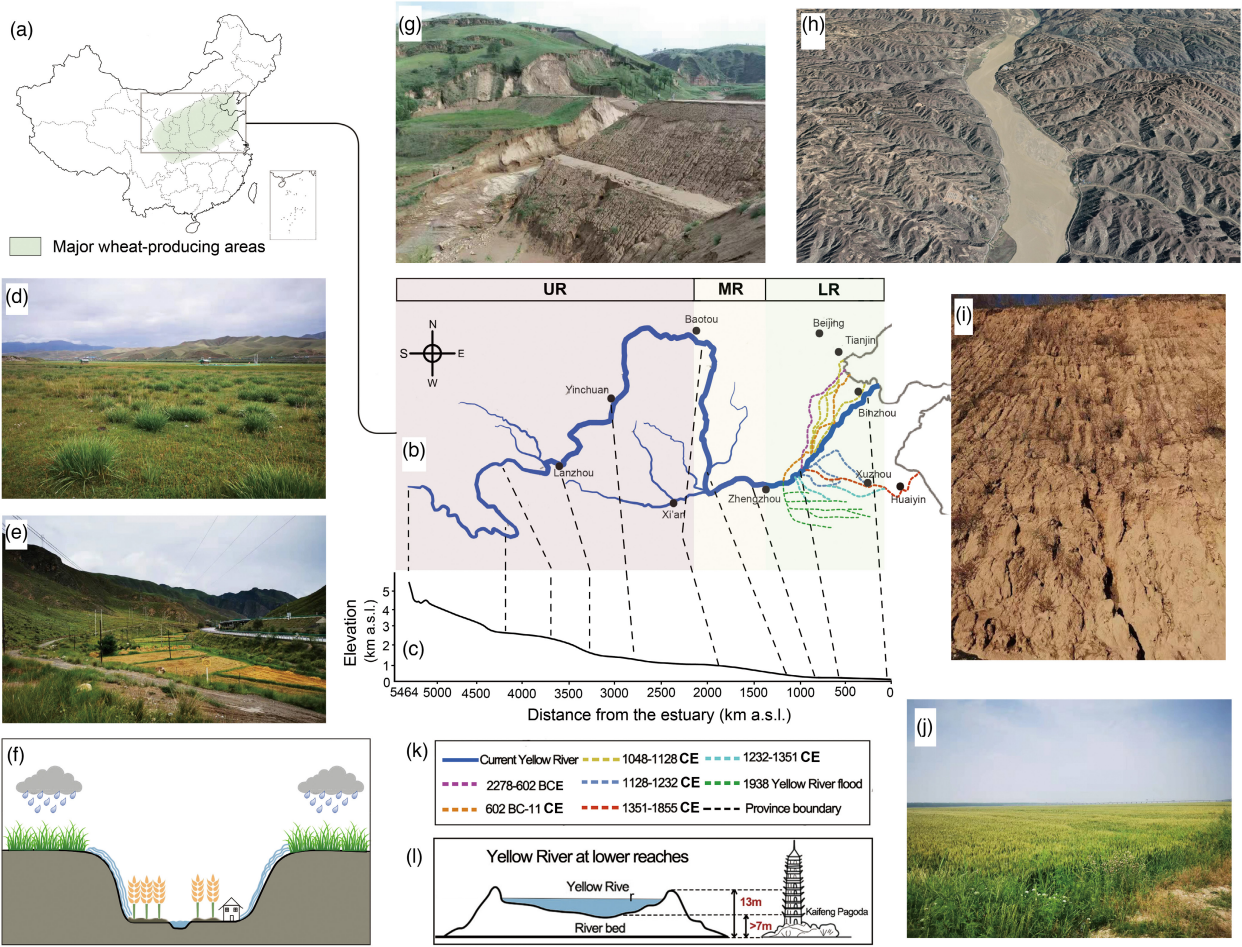
The landscapes and schematics of the Yellow River in its upper, middle and lower reaches. (a) Map of China with major wheat growing areas marked in green shadow. (b) Map of Yellow River Basin and the subdivision of reaches. Colored dashed lines indicate the historical river courses. LR, lower reaches; MR, middle reaches; UR, upper reaches. (c) Altitude distribution along river showing total elevation drops is minor in middle and lower reaches. (d) The typical alpine meadow landscape in upper reaches. (e) Pictures of agro‐pastoral zone in upper reaches showing the alpine meadow and adjacent wheat field. (f) Image and schematic of agro‐pastoral zone. The village and wheat fields are located in valleys next to streams. In the rainy season, surface runoff carries cysts from alpine meadow downward to wheat fields, then to stream, and finally flows to tributaries and Yellow River. (g–i) Typical landscape of loess plateau in the middle reaches, where substantial erosion takes place. The large amount of mud and sand discharged into the river. Since it is an important wheat growing region, this process brings initial cyst source to local wheat and outputs more cysts into the river. Image h is adapted from Google earth. (j) The landscape of wheat growing region in lower reaches. This region is characterized by a large plain that is frequently flooded by Yellow River. (k) Historical courses of Yellow River, with map showing in image b. (l) Schematic of “hanging river” at lower reaches. The silts received from the middle reaches form sediments in lower reaches, elevating the river bed. Excessive sediment deposits have raised the riverbed several meters above the surrounding ground. At Kaifeng of Henan Province, the Yellow River is more than 10 m above the ground level.

## 4 DISCUSSION

### 4.1 Species identity and population structure

Through country‐wide sampling, we demonstrated that CHA is widely distributed in China. Our morphological study is congruent with the observation by Subbotin (2015), in that populations with haplotype H1 (GenBank accession KU1K47195–KU147198) have smaller average cyst sizes in comparison to the typical EHA populations. Similar differences were also reported in pathotypes (Cui et al., 2015; Peng & Cook, 1996; Yuan et al., 2010; Zheng et al., 1997) and ITS‐RFLP profiles (Subbotin et al., 2003; Zheng et al., 2000). Subbotin (2015) proposed two mtCOI haplotypes (H1 and H7) for *H. sturhani* to accommodate all CHA. However, such assignment was not accepted by some researchers as they considered these differences insufficient to warrant a species rank redescription (Peng, Holgado, et al., 2016; Peng, Li, et al., 2016). In the present study, our molecular species delimitation supports *H. avenae* as one (including *H. arenaria*), two, and five species depending on the methods used, but none of them included CHA ( = *H. sturhani* proposed by Subbotin (2015)), suggesting that CHA indeed represents an independent evolutionary lineage different from that of the typical European *H. avenae* populations. That CHA and *H. pratensis* were reconstructed as the same species or clumped together with *H. australis* as one species, depending on the analysis. Although the close relationship between CHA and *H. pratensis* was previously known, they have been treated as two valid species and can be distinguished by unique morphometrics and host range, CHA has smaller cysts, parasitizes wheat, barley, wild and cultivated oat, whereas *H. pratensis* has larger cysts, presently known only as a parasite of grasses (Subbotin, 2015; Subbotin et al., 2010, 2018). However, our recovery of intermediate haplotypes (H1, H6, and H11) that can parasite both grass and wheat, and our finding that grass parasitic populations (H9 and H20) have smaller size, challenges the validation of CHA and *H. pratensis* as two distinct species. More recently, a similar study (Shao et al., 2022) sequenced 80 samples and recovered 41 haplotypes associated with wheat. Although both studies were conducted independently in two research groups using complete different samples, they showed very similar population structure patterns, except for EHA haplotypes were recovered in Shao et al. (2022) while only CHA were found in our study.

The molecular species‐delimitation analyses are known to generate a variety of different species hypotheses (Kekkonen & Hebert, 2014; Qing et al., 2019), and thus their outputs should be handled with caution. As a result, multiple lines of evidences are important to support species hypotheses. In Nematoda, morphology‐based species identification is challenging, as the phenotypic plasticity is rather common (Coomans, 2002; Nadler, 2002), and even the progeny of a single parthenogenetic female nematode can vary greatly in different environmental conditions (Fonderie et al., 2013). In respect to the *H. avenae* group, although the average cyst size trends to be interspecifically conserved, it is clear they overlap in range and differ among populations (Handoo, 2002; Subbotin et al., 2003). Unfortunately, it remains unclear whether such morphometrics differences are stable in different environmental conditions, e.g., temperature, moisture, soil texture, and host species, etc. Moreover, their genomic architecture, life cycle, and ecology of many nematode species are scarce or completely absent, thus species assignment using a single genetic marker or biological references is not possible. Therefore, we opted to follow the most stringent species criterion, by which a species is only recognized when it is supported by molecular phylogeny, morphological, and biological characters. Consequently, no species was proposed or synonymized in this study.

### 4.2 Speciation and historical distribution

Our biogeographic reconstruction indicates that northwest China is a center of *Heterodera* spp. diversification. Interestingly, this region is mostly covered by Qinghai‐Tibetan Plateau and has comparable geographic and climate condition to the Irano‐Anatolian Plateau, which is another *Heterodera* spp. speciation hotspot. Cyst nematodes are adapted to cold climate, and low temperature is necessary for their development (Evans & Stone, 1977; Smiley et al., 2017). Subbotin et al. (2018) noticed that mountain areas are possible regions for species diversification of cyst nematodes (*Globodera* spp. and *Heterodera* spp.). Therefore, the formation of Qinghai‐Tibetan Plateau and their local environment were likely the key for *Heterodera* spp. diversification.

The Qinghai‐Tibetan Plateau uplift throughout much of the Cenozoic (the last 66 million years) is one of the most important tectonic events in the Earth's history (Li et al., 2015). The average elevation of the plateau has gradually risen to over 4000 m.a.s.l. (Favre et al., 2015), creating the “Third Pole” that significantly influenced atmospheric circulation across the global mid‐latitudes and climates in adjacent regions (Nan et al., 2021). The formation of east and south Asian monsoons is an example of such influences (Sun & Liu, 2021), so was the aridification of Inner Asia (Li et al., 2018). By blocking moisture from the Pacific and Indian Oceans and disturbing the Westerlies, the uplifted plateau literally shaped up belts of deserts and grasslands across the heartland of the Eurasian continent (Barbolini et al., 2020). The initiation of that aridification dated to ca. 27 mya, when steppe vegetation started to appear in northwest China (Zhu et al., 2016). Although the geographical extent of this steppe is largely unknown, it is clear the formation of vast extent of steppe having provided various favorable monocotyledons hosts for the *Heterodera* spp. diversification. Indeed, our population genetic analysis revealed that the wheat *Heterodera* spp. in Qinghai‐Tibetan Plateau has much higher diversity in comparison to those from central China.

Over the last 8 million years, notably in the Quaternary (i.e., the last ca. 2.6 million years), global cooling resulted in an intensification of such aridity (Zhu et al., 2016). More permanent ice sheets started to appear in the high latitudes of the northern hemisphere and there have become alternating episodes of glacial and interglacial despite an overall trend of global cooling (Miao et al., 2017). This increasing level of aridity facilitated expansion of deserts and grasslands (Jia et al., 2020). The expansion of grasslands provided a highly favorable environment for the spread of *Heterodera* spp. On the other hand, frequent and high‐amplitude climatic oscillations between the glacials and interglacials might have acted as a key selection force that created fragmentary habitats for the diversification of *Heterodera* spp.

Following the formation of the Yellow River around ca. 0.2–0.1 mya (Wang et al., 2013), northwest China has been well connected to the Loess Plateau and the North China Plain. The CHA might have spread along the course of the Yellow River. However, in the warm and humid Holocene (the last ca. 11,700 years), when much of the North China Plain was covered by natural zonal vegetation of temperate forests (Li et al., 2019), at least along the lower reaches of the Yellow River, the distribution of CHA should have been highly constrained to the valleys of the river and its tributaries.

### 4.3 Role of Yellow River and machinery in dissemination

In this study, we demonstrated the speciation of CHA was a recent event and haplotype H1 was widespread across major wheat growing regions. Such results indicated an efficient transmission pathway was necessary. Nematodes can be transmitted in various manners, e.g., moving short distances on their own, by insect vectors, by rain or irrigation water, and by wind‐borne dust (Chizhov, 1996; McLeod, 1992; Ptatscheck et al., 2018; Siddiqi, 2000). Although these methods might be used serendipitously for cyst nematodes, they are unlikely to be the major forces that shaped their current distribution pattern.

Theoretically, river can transport various plant pathogens, but only in limited contribution to the disease distribution. In China, wheat growing and CHA occurrence areas are highly overlapping with the Yellow River (Figure 5a), thus its role in cysts transportation is suspected. The Yellow River is the second‐longest river in China and sixth‐longest in the world (5464 km). The source of the Yellow River is located in northeastern Qinghai‐Tibetan Plateau, where it is typically covered by alpine meadow (Figure 5d,e). Our study demonstrated CHA is widespread in this region, and the recovery of cysts in steams of valley suggested that matured cysts can flow into tributaries through rainwater and provide the initial cysts inoculums (Figure 5f). Afterward, the river flows through semiarid middle reaches where mean annual precipitation is only 400 mm for the whole drainage basin. In this stage, the Yellow River and its main tributaries drain the Loess Plateau, where substantial erosion takes place (Figure 5g–i). The Yellow River is considered as the most heavily silt‐laden river in the world (Leung, 1996). One estimate gives 34 kg of silt per cubic meter as opposed to 10 kg for the Colorado River and 1 kg for the Nile River (Tregear, 1965). In fact, the Loess Plateau contributes about 90% of the huge sediment load of this river (Ren & Shi, 1986). The drainage area of middle reaches consists of deep layer of favorite silty soil and has developed as main wheat growing region of China. However, the conflict of high irrigation demand and the low annually precipitation limited the wheat production and subsequently led to the extensive use of Yellow River for irrigation. An incomplete estimation suggests that irrigated areas within the basin as in total of more than 4 million ha, but nearly 2 million ha outside the basin also rely on water from the Yellow River (Cai et al., 2003). Although without direct support, it is logical to expect this irrigation process introduced cysts inoculum to wheat fields.

After the Loess Plateau, the Yellow River flows to lower reaches. Due to its heavy load of silt and low elevation drops (Figure 5c), the Yellow River flows slowly, deposits part of soil in its bed in stretches. As a result, excessive sediment deposits have raised riverbed several meters above the surrounding land (Leung, 1996) (Figure 5l). Historical records indicate progressively frequent levee breaching in the last 10 centuries. During such breaches, the flood water rushed onto the surrounding lands, not only inundating farmland and communities, but also taking over existing river channels. Records indicated that levees of the Yellow River had breached more than 1500 times and its course had changed 26 times in the last three millennia (Leung, 1996) (Figure 5b,k). These flooded areas overlapped with main wheat growing regions, including Henan, Anhui, Shandong, and the North part of Jiangsu Province. Interestingly, our survey indicated that CHA is widely distributed in the wheat fields of these regions, old courses of Yellow River, as well as in water flow of Yellow Rivers and its tributaries, and predominately in haplotype H1 (Figure 2). Indeed, similar result has already been noticed (Zhao et al., 2011). Therefore, the idea that frequent flood and changes of river channels can efficiently deliver cysts to wheat fields is supported, and consequently, the Yellow River is responsible for the long‐distance transportation of CHA populations and can be an important driver for current distribution pattern. In addition, we observed the higher haplotype diversity and genetic structure presented in the areas where the Yellow River never reached (e.g. TJ and BJ). These populations have similar genetic structure to grass‐parasitic *H. pratensis*, which may be a result of recent introduction from local grass hosts. It is known that nematodes can be carried by irrigation water (Hong & Moorman, 2005), but river‐mediated dissemination up to a few thousand kilometers was never reported in nematodes and rarely in other plant pathogens. In present study, although more data are needed to have conclusive result, we report for the first time that river can be a potential transmitter of nematode to an overwhelming scale that determines the country‐wide population distribution.

Farm machinery is also plausible for long‐distance dissemination. Wheat trans‐regional machinery harvesting only has a short history in China starting around the 2000s (Yang & Bai, 2000) and mainly in the plains in eastern China. Their key roles in cyst nematode transmission have been demonstrated in *H. filipjevi* (Madzhidov, 1981) Stelter, 1984. This species has relatively recognized history, which was probably introduced to China in the late 1950s, when the wheat cultivars from Albania were brought to China (Fu et al., 2011; Jin & Liu, 1964), and soon expanded to several wheat growing regions (Fu et al., 2011; Li et al., 2010; Peng et al., 2010, 2018; Peng, Holgado, et al., 2016; Peng, Li, et al., 2016; Zhen et al., 2018). Since no avulsion or flooding happened in Yellow River later than 1950s, the river contribution for the spreading of *H. filipjevi* was negligibly minor, thus supporting machinery as an important driver for dissemination. Similarly, we demonstrated that machinery can also carry cysts of CHA, suggesting that machinery is an important transmission approach in lower reaches of Yellow River, as it is in plain and trans‐regional machinery harvesting is common. This is in comparison to Northwest China, where wheat typically grows in mountainous regions and trans‐regional machinery harvesting is rare. Consequently, we expect that the impact of machinery mediated transmission is limited at middle and upper reaches.

### 4.4 Wheat cultivation and prevalence of H1 population

The Yellow River efficiently delivers various cysts haplotypes across its basin. However, the prevalence of haplotypes H1 population is related to the cultivation of wheat. The extensive monoculture of wheat sharply reduced the genetic variation of *Heterodera* spp. by natural selection of wheat‐preferred genotypes (e.g., haplotype H1). Through this population bottleneck, few superior genotypes were able to expand their populations rapidly and soon become predominant, which is similar to the process of Founder effect.

Recent findings (Long et al., 2018) pinpoint the spread of wheat to the heartland of China between ca. 5000 and 4000 BP or ca. 5200 BP on its northern border (Zhou et al., 2020), therefore the prevalence of CHA H1 population should be later than this time. Although the routes for its initial spread are still in question (Long et al., 2018), it might have been in the upper reaches of the Yellow River where wheat‐based agriculture was first intensified (Chen et al., 2019). Since the Han Dynasty (202 BCE–220 CE), wheat gradually replaced millets to become the staple crop in North China (Zhou et al., 2021). The H1 populations had the opportunity to spread by following establishment of wheat fields in the Loess Plateau and the North China Plain. The expansion scale of wheat fields and deforestation was unprecedented (Anderson, 2019), creating large areas of anthropogenic grasslands that were no longer constrained in river valleys. The distribution of H1 has been thus more extended than those of its predecessors.

## ACKNOWLEDGEMENT

This research was supported by the National Natural Science Foundation of China (Grant numbers 31471751 and 32001876) and the Special Fund for Agro‐scientific Research in the Public Interest (Grant numbers 200903040 and 201503114). TL acknowledges the Zhejiang Provincial Qianjiang Talent Scheme (Grant number QJC2002001). We thank Drs. Yulong Li and Gaofeng Wang for their assistance in sampling or providing samples. Prof. Fengbao Zhang is also acknowledged for providing photos of the Loess Plateau.

## CONFLICT OF INTEREST

The authors declare no competing interest.

## Supporting information


Appendix S1–S4

## Data Availability

The data that support the findings of this study are available from the corresponding author upon reasonable request.
