# Supplementary material for: Phylogeography of Chinese cereal cyst nematodes sheds lights on their origin and dispersal
Source: Evol Appl. 2022 Aug 4;15(8):1236–48. doi: 10.1111/eva.13452 (PMC9423084; doi:10.1111/eva.13452)
Supplement: Supplementary file 1 — Appendix S1–S4 [file EVA-15-1236-s001.pdf]

Supplementary Materials for

Phylogeography of Chinese cereal cyst nematodes sheds lights on their origin and dispersal

Xue Qing, Huan Peng, Jukui Ma, Y. Miles Zhang, Hongmei Li,* Deliang Peng,* Xuan Wang,* Tengwen Long

*Corresponding authors, Emails: lihm@njau.edu.cn, dlpeng@ippcaas.cn, xuanwang@njau.edu.cn,

Part 1: Sampling information

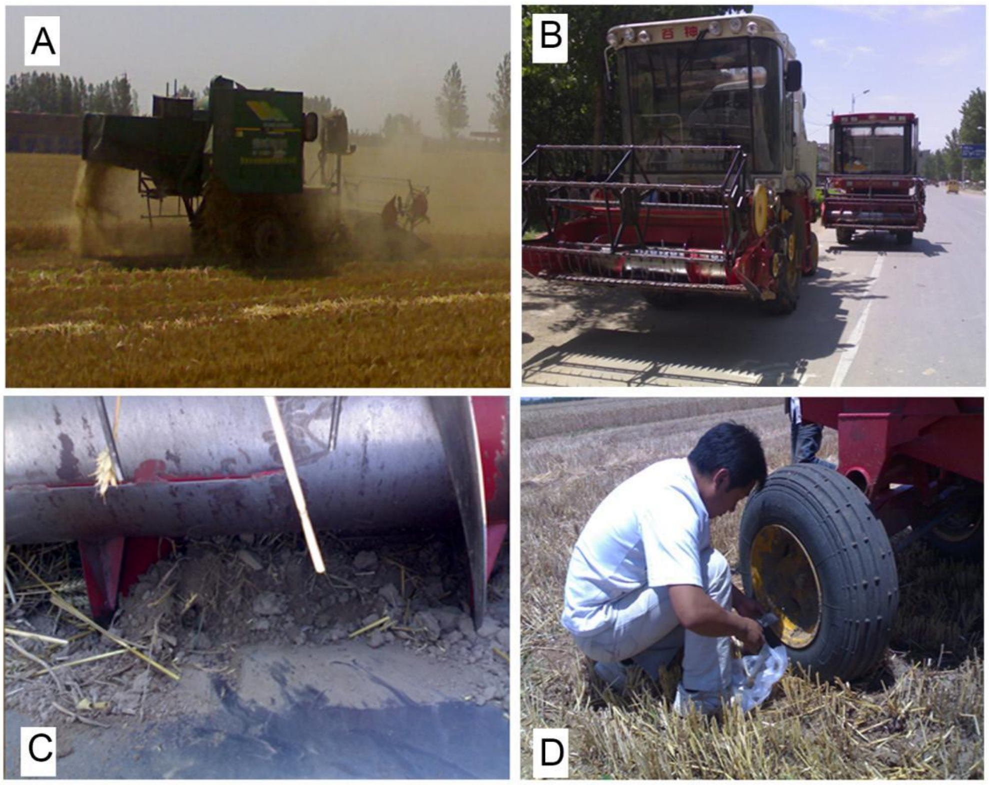

Fig. S1. The survey of cereal cyst nematodes in the soil attached to harvester machine. (**A** and **B**) Typical machinery harvester used in Hebei and Henan provinces of China. (**C** and **D**) Sampling of soil attached to reels and wheels of harvester machine.

Table S1. The positive sampling locations and corresponding recovered cyst densities. CD = cysts number per 100 g soil. Ab = abbreviations for the sampling provinces and locations. Abbreviations: JSXZ = Xuzhou, Jiangsu; JSTC = Taicang, Jiangsu; JSNT = Nantong, Jiangsu; HN = Henan, SD = Shandong; AH = Anhui; HD = Hebei; SX = Shanxi; SS = Shaanxi; GS = Gansu; QH = Qinghai; BJ = Beijing; TJ = Tianjin; NM = Neimeng; NX = Ningxia; HB = Hubei; TZ = Tianzhu; OYR = old courses of Yellow River. Asterisk (*) donate the sites failed to acquire mtCOI.

No.	Sampling locality	Latitude	Longitude	CN	Ab
1	Zhangjizhen, Tongshan, Xuzhou, Jiangsu	34.2421	117.5826	16.0	
2	Fangcun, Tongshan, Xuzhou, Jiangsu	34.1206	117.7842	13.7	
3	Liuji, Tongshan, Xuzhou, Jiangsu	34.6065	117.0234	7.0	
4	Liuji, Tongshan, Xuzhou, Jiangsu*	34.6082	117.0013	17.0	
5	Liuji, Tongshan, Xuzhou, Jiangsu	34.6614	117.6156	24.0	
6	Liuji, Tongshan, Xuzhou, Jiangsu	34.6636	117.5997	33.3	
7	Heqiao, Tongshan, Xuzhou, Jiangsu	34.7483	117.5304	8.0	
8	Heqiao, Tongshan, Xuzhou, Jiangsu	34.3342	117.0253	45.0	
9	Heqiao, Tongshan, Xuzhou, Jiangsu	34.7710	117.3648	9.3	
10	Huangji, Tongshan, Xuzhou, Jiangsu	34.3926	116.9520	73.7	
11	Liuji, Tongshan, Xuzhou, Jiangsu	35.1082	117.5301	4.0	
12	Dashahe, Fengxian, Xuzhou, Jiangsu	35.1095	117.1234	8.3	
13	Huashan, Fengxian, Xuzhou, Jiangsu	35.0849	117.1556	3.0	
14	Huashan, Fengxian, Xuzhou, Jiangsu	35.0407	117.2444	2.5	
15	Fanlou, Fengxian, Xuzhou, Jiangsu	34.7888	117.4303	30.3	
16	Hekou, Fengxian, Xuzhou, Jiangsu	34.9803	117.3286	13.7	
17	Qishan, Fengxian, Xuzhou, Jiangsu	35.0946	117.3485	12.3	
18	Qishan, Fengxian, Xuzhou, Jiangsu	35.1461	117.3520	17.3	
19	Changdian, Fengxian, Xuzhou, Jiangsu	35.2061	116.8650	11.0	
20	Zhaozhuang, Fengxian, Xuzhou, Jiangsu	35.2159	116.8136	15.0	JSXZ
21	Zhaozhuang, Fengxian, Xuzhou, Jiangsu	35.2320	116.7126	9.7	
22	Wanggou, Fengxian, Xuzhou, Jiangsu	35.0413	116.7473	16.3	
23	Wanggou, Fengxian, Xuzhou, Jiangsu	35.0888	116.7567	35.7	
24	Songlou, Fengxian, Xuzhou, Jiangsu	35.0140	116.8334	81.3	
25	Liangzhai, Fengxian, Xuzhou, Jiangsu	35.0792	116.9498	1.7	
26	Liangzhai, Fengxian, Xuzhou, Jiangsu	35.1624	116.9791	3.7	
27	Xuzhuang, Fengxian, Xuzhou, Jiangsu	34.3018	117.4629	27.0	
28	Xuzhuang, Fengxian, Xuzhou, Jiangsu	34.4730	117.3184	34.3	
29	Liuzhuang, Fengxian, Xuzhou, Jiangsu	35.5091	117.3668	4.0	
30	Xiaolizhuang, Fengxian, Xuzhou, Jiangsu	35.0140	116.8334	4.7	
31	Hekou, Peixian, Xuzhou, Jiangsu	34.9949	117.3003	15.3	
32	Jingan, Peixian, Xuzhou, Jiangsu	34.8860	117.4353	60.0	
33	Lulou, Peixian, Xuzhou, Jiangsu	35.2348	117.2042	10.0	
34	Lulou, Peixian, Xuzhou, Jiangsu	35.3037	117.1677	9.3	
35	Shuanggou, Zining, Xuzhou, Jiangsu	34.0385	118.0663	32.0	
36	Wangji, Zining, Xuzhou, Jiangsu	34.6263	118.2320	10.3	
37	Lanshan, Zining, Xuzhou, Jiangsu	34.5122	118.2148	54.3	
38	Taoyuan, Zining, Xuzhou, Jiangsu	34.3944	118.3019	32.0	
39	Zhuzhai, Peixian, Xuzhou, Jiangsu	34.7335	116.8119	3.0	

(continue)

No.	Sampling locality	Latitude	Longitude	CN	Ab
40	Zhuzhai, Peixian, Xuzhou, Jiangsu	34.7564	116.8317	37.7	
41	Yuanyanglou, Peixian.Xuzhou, Jiangsu	34.7827	116.7180	23.7	
42	Fengxian, Xuzhou, Jiangsu	34.7103	116.6994	6.0	
43	Fengxian, Xuzhou, Jiangsu	34.7103	116.6994	14.3	
44	Fengxian, Xuzhou, Jiangsu*	34.6776	166.6707	27.7	
45	Tongshan, Xuzhou, Jiangsu	34.6712	116.6537	53.7	
46	Tongshan, Xuzhou, Jiangsu	34.6467	116.7001	9.3	
47	Tongshan, Xuzhou, Jiangsu	34.0729	117.5029	9.7	
48	Zining, Xuzhou, Jiangsu	33.9309	117.8041	43.0	
49	Zining, Xuzhou, Jiangsu	33.9316	117.7472	16.3	
50	Zining, Xuzhou, Jiangsu*	33.9899	117.7829	8.3	JSXZ
51	Pizhou, Xuzhou, Jiangsu	34.4367	117.9596	3.0	
52	Pizhou, Xuzhou, Jiangsu	34.4657	117.9492	0.7	
53	Pizhou, Xuzhou, Jiangsu	34.4685	117.9626	6.3	
54	Xiyi, Xuzhou, Jiangsu	34.2891	118.2845	2.3	
55	Xinyi, Xuzhou, Jiangsu	34.2891	118.2845	66.3	
56	Longhe, Suqian, Jiangsu	33.8666	118.2583	2.0	
57	Siyang, Suqian, Jiangsu	33.5588	118.3678	33.3	
58	Siyang, Suqian, Jiangsu*	33.6875	118.6284	1.0	
59	Siyang, Suqian, Jiangsu	33.7507	118.6284	25.3	
60	Siyang, Suqian, Jiangsu	33.8673	118.7365	33.7	
61	Siyang, Suqian, Jiangsu	33.9583	118.7253	1.7	
62	Tonglibeilian, Wujiang, Suzhou, Jiangsu	31.2879	121.2008	3.2	
63	Yangshe, Zhangjiagang, Suzhou, Jiangsu	31.9288	120.5206	11.0	
64	Changyinsha, Zhangjiagang, Suzhou, Jiangsu	31.8791	120.7845	5.3	
65	Guli, Changsu, Suzhou, Jiangsu	31.5818	120.8730	2.8	JSTC
66	Shuangfengtangdong, Taicang, Suzhou, Jiangsu	31.5255	121.0468	68.3	
67	Huangjing, Taicang, Suzhou, Jiangsu	31.6888	121.0587	2.7	
68	Yushan, Kunshan, Suzhou, Jiangsu	31.3556	120.8873	3.0	
69	Qutang, Haian, Nantong, Jiangsu	32.9928	121.3738	12.0	
70	Qutang, Haian, Nantong, Jiangsu	32.5569	120.4408	3.3	
71	Jiaoxie, Haian, Nantong, Jiangsu	32.5569	120.4408	79.3	
72	Jiaoxie, Haian, Nantong, Jiangsu	32.5564	120.4401	4.7	
73	Jiaoxie, Haian, Nantong, Jiangsu	32.5564	120.4399	1.3	
74	Huji, Haian, Nantong, Jiangsu	32.5564	120.4398	21.7	JSNT
75	Huji, Haian, Nantong, Jiangsu	32.5693	120.7766	2.3	
76	Yazhou, Haian, Nantong, Jiangsu	32.5693	120.7761	7.7	
77	Laobagang, Haian, Nantong, Jiangsu	32.5693	120.7757	2.4	
78	Laobagang, Haian, Nantong, Jiangsu	32.5693	120.7764	4.3	
79	Laobagang, Haian, Nantong, Jiangsu	32.5693	120.7754	15.0	

(continue)

No.	Sampling locality	Latitude	Longitude	CN	Ab
80	Yazhou, Haian, Nantong, Jiangsu	32.5693	120.7752	255.0	JSNT
81	Yazhou, Haian, Nantong, Jiangsu	32.5693	120.7748	95.0	
82	Sunzhuang, Haian, Nantong, Jiangsu	32.4124	120.3952	7.0	
83	Sunzhuang, Haian, Nantong, Jiangsu	32.4123	120.3951	51.3	
84	Sunzhuang, Haian, Nantong, Jiangsu	32.4123	120.3950	39.7	
85	Sunzhuang, Haian, Nantong, Jiangsu*	32.4167	120.3929	7.3	
86	Sunzhuang, Haian, Nantong, Jiangsu	32.4219	120.3218	5.3	
87	Sunzhuang, Haian, Nantong, Jiangsu	32.4219	120.3200	9.7	
88	Wuyou, Tinghu, Yancheng, Jiangsu	33.4392	120.2698	1.7	
89	Louwang, Yandu, Yancheng, Jiangsu	33.5059	120.3632	202.0	
90	Jiangan, Rugao, Nantong, Jiangsu	32.1367	120.4373	2.7	
91	Banjing, Rugao, Nantong, Jiangsu	32.3400	120.4561	14.3	
92	Shigang, Tongzhou, Nantong, Jiangsu	32.1861	120.9601	5.0	
93	Matang, Rudong, Nantong, Jiangsu	32.3250	121.0694	3.7	
94	Huanzhen, Nantong, Jiangsu	32.3201	121.1391	5.0	
95	Laobagang, Haian, Nantong, Jiangsu	32.5957	120.8243	1.3	
96	Jiaoling, Chuzhou, Huaian, Jiangsu	33.7232	119.3084	95.0	
97	Jiaoling, Chuzhou, Huaian, Jiangsu	33.6920	119.3356	7.3	
98	Shunhe, Chuzhou, Huaian, Jiangsu	33.6832	119.3466	1.0	
99	Shunhe, Chuzhou, Huaian, Jiangsu*	33.6095	119.2642	49.7	
100	Jiqiao, Chuzhou, Huaian, Jiangsu	33.6062	119.2555	9.7	
101	Jiqiao, Chuzhou, Huaian, Jiangsu	33.6062	119.2555	4.7	
102	Jiqiao, Chuzhou, Huaian, Jiangsu	33.5596	119.2174	20.0	
103	Sanbao, Chuzhou, Huaian, Jiangsu	33.4579	119.1337	2.3	
104	Sanbao, Chuzhou, Huaian, Jiangsu	33.4579	119.1337	1.3	
105	Fanji, Chuzhou, Huaian, Jiangsu	33.3703	119.0773	66.3	
106	Zhuba, Hongze, Huaian, Jiangsu	33.2983	118.9332	8.7	
107	Chahe, Hongze, Huaian, Jiangsu	33.2556	119.0052	1.0	
108	Qianfeng, Jinhu, Huaian, Jiangsu	33.1140	119.1436	37.0	
109	Maba, Xuyi, Huaian, Jiangsu	32.9392	118.7754	2.7	
110	Fanchuan, Jiangdu, Yangzhou, Jiangsi	33.0094	120.1321	21.0	
111	Xiannv, Jiangdu, Yangzhou, Jiangsi	32.6024	120.1919	12.0	
112	XiannvJiangdu, Yangzhou, Jiangsi	32.6115	120.2791	27.3	
113	Shaobo, Jiangdu, Yangzhou, Jiangsi	32.6091	120.2009	26.3	
114	Daqiao, Jiangdu, Yangzhou, Jiangsu	32.3655	119.7206	3.7	
115	Fanchuan, Jiangdu, Yangzhou, Jiangsu	32.6056	119.6792	16.3	
116	Hanliu, Gaoyou, Yangzhou, Jiangsu	32.7120	119.6751	12.3	
117	Sanduo, Gaoyou, Yangzhou, Jiangsu	32.8234	119.6731	4.7	
118	Longqiu, Gaoyou, Yangzhou, Jiangsu	32.8064	119.5052	8.0	
119	Longqiu, Gaoyou, Yangzhou, Jiangsu	32.9087	119.5047	46.0	

(continue)

No.	Sampling locality	Latitude	Longitude	CN	Ab
120	Zhouwang, Gaoyou, Yangzhou, Jiangsu	32.9466	119.5045	18.0	JSNT
121	Zhouxiang, Gaoyou, Yangzhou, Jiangsu	33.0198	119.5043	12.3	
122	Fanshui, Baoying, Yangzhou, Jiangsu	33.1112	119.4093	7.7	
123	Xiaoguanzhuang, Baoying, Yangzhou, Jiangsu	33.1956	119.3877	51.0	
124	Anyi, Baoying, Yangzhou, Jiangsu	33.1964	119.3481	7.0	
125	Wangzhigang, Baoying, Yangzhou, Jiangsu	33.2463	119.4123	6.3	
126	Liubao, Baoying, Yangzhou, Jiangsu	33.1431	119.5217	8.0	
127	Xiaji, Baoying, Yangzhou, Jiangsu	33.0983	119.5469	13.3	
128	Zhongbao, Xinghua, Taizhou, Jiangsu	33.0877	119.8555	20.3	
129	Lincheng, Xinghua, Taizhou, Jiangsu	32.8594	119.8610	3.7	
130	Liulu, Xinghua, Taizhou, Jiangsu	32.8323	119.8804	1.0	
131	Liulu, Xinghua, Taizhou, Jiangsu	32.8262	119.9178	0.7	
132	Xingtai, Jiangyan, Taizhou, Jiangsu*	32.6816	120.0961	4.3	
133	Xingtai, Jiangyan, Taizhou, Jiangsu	32.6799	120.0961	3.0	
134	Shengao, Jiangyan, Taizhou, Jiangsu	32.5457	120.1033	2.0	
135	Liangxu, Jiangyan, Taizhou, Jiangsu*	32.4520	120.1491	28.0	
136	Liangxu, Jiangyan, Taizhou, Jiangsu	32.4301	120.0918	1.0	
137	Zhangdian, Jiangyan, Taizhou, Jiangsu	32.4079	120.0750	10.0	
138	Gugao, Jiangyan, Taizhou, Jiangsu	32.3655	120.1315	26.7	
139	Jichuan, Taixing, Taizhou, Jiangsu	32.4751	120.3507	142.3	
140	Yuanzhu, Taixing, Taizhou, Jiangsu	32.3472	120.1708	80.7	
141	Huangqiao, Taixing, Taizhou, Jiangsu	32.2851	120.2104	12.7	
142	Heshi, Taixing, Taizhou, Jiangsu	32.1985	120.1151	11.7	
143	Zhangqiao, Taixing, Taizhou, Jiangsu	32.1052	120.0715	17.7	
144	Bilu, Jingjiang, Taizhou, Jiangsu	32.0745	120.0952	8.0	
145	Shengci, Jingjiang, Taizhou, Jiangsu	32.0498	120.1374	26.3	
146	Maqiao, Jingjiang, Taizhou, Jiangsu	32.0409	120.1904	6.3	
147	Xieqiao, Jingjiang, Taizhou, Jiangsu	32.0399	120.3428	8.3	
148	Xilai, Jingjiang, Taizhou, Jiangsu	32.1033	120.4186	26.3	
149	Wuyou, Tinghu, Yancheng, Jiangsu	33.2811	120.2441	6.7	
150	Guomeng, Yandu, Yancheng, Jiangsu	33.2593	120.0698	0.3	
151	Yandan, Jianhu, Yancheng, Jiangsu	33.3537	119.7371	1.0	
152	Jinhu, Jianhu, Yancheng, Jiangsu	33.4194	119.7669	2.0	
153	Zhongzhuang, Jianhu, Yancheng, Jiangsu	33.5328	119.8515	3.0	
154	Fucheng, , Funing, Yancheng, Jiangsu	33.7446	119.7720	12.3	
155	Shuoji, Funing, Yancheng, Jiangsu	33.6854	119.6734	14.7	
156	Banhu, Funing, Yancheng, Jiangsu	33.6795	119.6344	7.3	
157	Tongyu, Binhai, Yancheng, Jiangsu	33.9501	119.8552	14.7	
158	Zhenghong, Binhai, Yancheng, Jiangsu	33.8824	119.8604	1.0	
159	Zhenghong, Binhai, Yancheng, Jiangsu	33.8497	119.8269	5.3	

(continue)

No.	Sampling locality	Latitude	Longitude	CN	Ab
160	Liutao, XiangshuiYancheng, Jiangsu	34.1116	119.7880	8.0	JSNT
161	Qitao, Xiangshui, Yancheng, Jiangsu	34.1386	119.8210	2.3	
162	Lianyungang, Jiangsu	34.2092	119.2260	2.3	
163	Shahe, Ganyu, Lianyungang, Jiangsu	34.7262	118.9311	1.0	
164	Shilianghe, Ganyu, Lianyungang, Jiangsu*	34.7648	118.9248	1.0	
165	Hongzhuang, Donghai, Lianyungang, Jiangsu	34.4921	118.5975	1.0	
166	Jialou, Xuzhou, Jiangsu	34.3143	116.9813	2.5	OYR
167	Jialou, Xuzhou, Jiangsu	34.3143	116.9813	3.0	
168	Dapeng, Xuzhou, Jiangsu	34.2678	117.0417	11.3	
169	Dapeng, Xuzhou, Jiangsu	34.2512	117.0668	5.7	
170	Dapeng, Xuzhou, Jiangsu	34.2326	117.0416	2.0	
171	Dapeng, Tongshan, Xuzhou, Jiangsu	34.2326	117.0416	2.0	
172	Dapeng, Tongshan, Xuzhou, Jiangsu	34.2561	117.0809	3.0	
173	Sanbao, Tongshan, Xuzhou, Jiangsu	34.0902	117.1964	12.0	
174	Xiaoxian, Suzhou, Anhui	34.1164	117.1250	3.0	
175	Tangzhang, Xuzhou, Jiangsu	34.1017	117.2605	2.5	
176	Tangzhang, Xuzhou, Jiangsu	34.1017	117.2605	16.0	
177	Shuyang, Suqian, Jiangsu	34.1482	118.7359	5.3	
178	Shuyang, Suqian, Jiangsu	34.1482	118.7359	2.3	
179	Shuyang, Suqian, Jiangsu	34.1482	118.7359	1.0	
180	Shuyang, Suqian, Jiangsu	34.1489	118.7418	32.3	
181	Shuyang, Suqian, Jiangsu	34.1608	118.8348	22.3	
182	Shuyang, Suqian, Jiangsu	34.1526	118.8603	20.0	
183	Shuyang, Suqian, Jiangsu	34.1526	118.8603	15.3	
184	Guannan, Lianyungang, Jiangsu	34.1977	119.2252	42.5	
185	Guannan, Lianyungang, Jiangsu	34.1977	119.2252	35.0	
186	Chenguanzhuang, Yongcheng, Shangqiu, Henan	34.0275	116.9822	88.0	HN
187	Huicun, Yongcheng, Shangqiu, Henan	34.6310	116.8634	53.0	
188	Shibali, Yongcheng, Shangqiu, Henan	34.5862	116.5631	89.0	
189	Zanyang, Yongcheng, Shangqiu, Henan	34.6610	116.3971	102.3	
190	Mamu, Yongcheng, Shangqiu, Henan	34.0651	116.2936	36.3	
191	Tiaohezhongzai, Yongcheng, Shangqiu, Henan	34.4834	116.8321	60.0	
192	Mangshan, Yongcheng, Shangqiu, Henan	34.3403	116.8398	7.3	
193	Mangshan, Yongcheng, Shangqiu, Henan	34.2701	116.7945	56.0	
194	Xuehu, Yongcheng, Shangqiu, Henan	34.2447	116.7667	7.7	
195	Xuehu, Yongcheng, Shangqiu, Henan	34.1573	116.7465	14.0	
196	Chenji, Yongcheng, Shangqiu, Henan	34.0482	116.7017	8.0	
197	Shibali, Yongcheng, Shangqiu, Henan	34.5861	116.5633	19.0	
198	Shibali, Yongcheng, Shangqiu, Henan	34.5931	116.5479	15.7	
199	Shibali, Yongcheng, Shangqiu, Henan	34.6135	116.5031	5.0	

(continue)

No.	Sampling locality	Latitude	Longitude	CN	Ab
200	Zanlou, Yongcheng, Shangqiu, Henan	34.6112	116.3989	11.0	HN
201	Zimu, Yongcheng, Shangqiu, Henan	33.0421	116.3103	12.7	
202	Huiting, Xiayi, Shangqiu, Henan	34.1318	116.1601	75.3	
203	Jiyang, Xiayi, Shangqiu, Henan	34.2826	116.5947	30.7	
204	Huiting, Xiayi, Shangqiu, Henan	34.1321	116.1603	2.3	
205	Guodian, Xiayi, Shangqiu, Henan	34.2384	116.1507	35.7	
206	Mengdaqiao, Xiayi, Shangqiu, Henan	34.3150	116.1852	135.3	
207	Zhanji, Yucheng, Shangqiu, Henan	34.3655	116.4660	191.7	
208	Gushu, Yucheng, Shangqiu, Henan	34.4236	116.3411	118.0	
209	Mangzhongqiao, Yucheng, Shangqiu, Henan	34.5173	116.1774	12.7	
210	Wangmeng, Linying, Luohe, Henan	33.7896	113.9610	10.7	
211	Wangmeng, Linying, Luohe, Henan	33.7947	113.9859	18.3	
212	Wangmeng, Linying, Luohe, Henan	33.7887	114.0002	42.3	
213	Wangmeng, Linying, Luohe, Henan	33.7910	113.9794	40.0	
214	Wangmeng, Linying, Luohe, Henan	33.7865	113.9077	7.0	
215	Wangmeng, Linying, Luohe, Henan	33.7903	113.9694	14.3	
216	Wangmeng, Linying, Luohe, Henan	33.7960	113.9746	1.7	
217	Wangmeng, Linying, Luohe, Henan	33.7909	114.5817	4.7	
218	Wangmeng, Linying, Luohe, Henan	33.7973	114.0019	1.3	
219	Wangmeng, Linying, Luohe, Henan	33.7990	114.6077	4.0	
220	Wangmeng, Linying, Luohe, Henan	33.7898	114.6023	2.3	
221	Jiangguanchi, Xuchang, Henan	33.9715	113.8062	34.3	
222	Jiangguanchi, Xuchang, Henan	33.9409	113.7890	4.0	
223	Jiangguanchi, Xuchang, Henan	33.9435	113.7688	4.7	
224	Jiangguanchi, Xuchang, Henan	33.9391	113.7220	22.3	
225	Jiangguanchi, Xuchang, Henan	33.9545	113.7328	10.7	
226	Jiangguanchi, Xuchang, Henan	33.9749	113.7552	11.0	
227	Balitun, Weihui, Xinxiang, Henan	34.7490	114.1088	52.0	
228	Dunfangdian, Weihui, Xinxiang, Henan	35.8239	114.1556	25.7	
229	Shipogu, Yanjin, Xinxiang, Henan	35.4447	114.3764	108.0	
230	Yulin, Yanjin, Xinxiang, Henan	35.1971	114.0843	47.7	
231	Yuin, Yanjin, Xinxiang, Henan	35.1018	114.0156	50.0	
232	Ayang, Yuanyang, Xinxiang, Henan	35.1203	113.8842	54.3	
233	Qitang, Yuanyang, Xinxiang, Henan	35.4164	113.2963	49.3	
234	Chengguan, Yuanyang, Xinxiang, Henan	35.3715	114.0350	87.0	
235	Guguzhai, Xinxiang, Henan	35.3230	114.1268	23.0	
236	Guguzhai, Xinxiang, Henan	35.2707	114.1085	9.3	
237	Beiyang, Qixian, Hebi, Henan	35.9271	114.2267	21.0	
238	Gaocun, Qixian, Hebi, Henan	36.1718	114.4029	12.0	
239	Juqiao, Qixian, Hebi, Henan	36.1620	114.5116	86.3	

(continue)

No.	Sampling locality	Latitude	Longitude	CN	Ab
240	Youlicheng, Tangyin, Anyang, Henan	36.5910	114.5724	40.3	HN
241	Zhuangying, Huaxian, Anyang, Henan	36.0790	114.9333	50.3	
242	Wadian, Anyang, Henan	35.8589	115.2277	37.7	
243	Jindegu, Nanle, Puyang, Henan	36.1425	115.2275	30.0	
244	Chengguan, Nanle, Puyang, Henan	36.1021	115.2624	32.7	
245	Fanbao, Qingfeng, Puyang, Henan	36.6443	115.2941	33.3	
246	Shaiguzhuang, Qingfeng, Puyang, Henan	36.5620	115.2320	45.3	
247	Liuge, Qingfeng, Puyang, Henan	36.3937	115.1938	38.3	
248	Qinghetou, Puyangxian, Puyang, Henan	36.2187	115.1689	12.3	
249	Dongmaying, Boai, Jiaozuo, Henan	35.2253	113.1439	14.0	
250	Qinghua, Boai, Jiaozuo, Henan	35.2897	113.1984	56.3	
251	Xitielu, Jiaozuo, Henan	35.3509	113.2604	24.3	
252	Zhoukou, Henan	33.1378	113.2228	130.7	
253	Qingguji, Caoxian, Heze, Shandong	35.0825	116.2775	9.7	SD
254	Qingguji, Caoxian, Heze, Shandong	35.1539	116.3606	55.7	
255	Guocun, Shanxian, Heze, Shandong	35.2416	116.5909	7.7	
256	Shilipu, Shanxian, Heze, Shandong	35.4245	116.1931	3.3	
257	Zhongxing, Shanxian, Heze, Shandong	35.2371	116.5835	11.7	
258	Zhuji, Shanxian, Heze, Shandong	35.1303	116.5869	44.7	
259	Daokoupu, Dongchangfu, Liaocheng, Shandong	36.8106	116.3618	172.3	
260	Yuji, Dongchangfu, Liaocheng, Shandong	36.3888	116.6633	18.7	
261	Dingyuanzhai, Guanxian, Liaocheng, Shandong	36.5110	116.0179	97.7	
262	Sangazhen, Guanxian, Liaocheng, Shandong	36.7700	116.0962	9.7	
263	Liangtangzhen, Guanxian, Liaocheng, Shandong	36.7204	116.0522	45.0	
264	Dawangzhai, Shenxian, Liaocheng, Shandong	36.7078	115.8282	19.0	
265	Dongduzhuang, Shenxian, Liaocheng, Shandong	36.4634	115.8574	13.0	
266	Zhangzai, Shenxian, Liaocheng, Shandong	36.2226	115.8310	21.0	
267	Zhangzai, Shenxian, Liaocheng, Shandong	36.1297	115.8280	38.0	
268	Xihu, Yanggu, Liaocheng, Shandong	36.1025	115.8818	36.3	
269	Chengguan, Yanggu, Liaocheng, Shandong	36.2741	116.1976	70.3	
270	Huarunqiao, Yanggu, Liaocheng, Shandong	36.2434	116.2835	64.7	
271	Anle, Yanggu, Liaocheng, Shandong	36.2451	116.4176	24.3	
272	Qiji, Yanggu, Liaocheng, Shandong	36.2864	116.4566	20.7	
273	Damiao, Lingbi, Suzhou, Anhui	34.2743	118.1299	24.0	AH
274	Dalu, Lingbi, Suzhou, Anhui	34.3177	117.6368	10.0	
275	Huigou, Lingbi, Suzhou, Anhui	34.2447	117.6424	8.0	
276	Yinji, Lingbi, Suzhou, Anhui	34.3062	117.7695	20.3	
277	Xialou, Lingbi, Suzhou, Anhui	34.6440	117.7996	8.3	
278	Tubai, Xiaoxian, Suzhou, Anhui	34.2293	117.0935	75.7	
279	Dulou, Xiaoxian, Suzhou, Anhui	34.3462	117.4132	47.7	

(continue)

No.	Sampling locality	Latitude	Longitude	CN	Ab
280	Wangzai, Xiaoxian, Suzhou, Anhui	34.2606	117.2734	76.7	AH
281	Zulou, Xiaoxian, Suzhou, Anhui	34.1194	117.1639	5.7	
282	Liuanlou, Dangshan, Suzhou, Anhui	35.0256	116.6960	16.0	
283	Huaguanying, Cixian, Handan, Hebei	36.8246	114.7223	44.7	HD
284	Matou, Cixian, Handan, Hebei	36.7530	114.6553	15.3	
285	Zhangliji, Linzhang, Handan, Hebei	36.5882	114.7680	43.7	
286	Linzhangzhen, Linzhang, Handan, Hebei	36.5302	114.9685	7.3	
287	Xiyanggao, Linzhang, Handan, Hebei	36.5683	115.1566	41.3	
288	Yuanbao, Weixian, Handan, Hebei	36.5584	115.3506	63.3	
289	Shakou, Weixian, Handan, Hebei	36.8309	115.0080	84.0	
290	Shuangjing, Weixian, Handan, Hebei	36.6087	115.1441	13.0	
291	Shengying, Guangping, Handan, Hebei	36.5474	115.0345	114.3	
292	Zhanggu, Guangping, Handan, Hebei	36.5139	115.1273	38.7	
293	Zhanghedian, Chengan, Handan, Hebei	36.4413	115.6199	11.3	
294	Ghenganzhen, Chengan, Handan, Hebei	36.6398	115.4356	48.3	
295	Hesha, Handanxian, Handan, Hebei	36.7802	115.6086	102.0	
296	Lizhuang, Daming, Handan, Hebei	36.6793	115.3728	76.7	
297	Xiweizhuang, Daming, Handan, Hebei	36.7181	115.2045	17.7	
298	Jiudian, Feixiang, Handan, Hebei	36.9506	115.5940	20.0	
299	Anzhai, Zhouqu, Handan, Hebei	37.1633	115.5957	43.7	
300	Quzhou, Zhouqu, Handan, Hebei	37.2602	115.5384	2.7	
301	Caozhuang, Jize, Handan, Hebei	37.2790	115.4845	81.7	
302	Tongcheng, Wenxi, Yuncheng, Shanxi	35.6034	111.4242	1.7	SX
303	Guojiazhuang, Wenxi, Yuncheng, Shanxi	35.5290	111.2915	12.7	
304	Wenxi, Yuncheng, Shanxi	35.5807	111.3062	44.0	
305	Wadi, Wenxi, Yuncheng, Shanxi	35.7578	111.1968	5.3	
306	Liyuan, Wenxi, Yuncheng, Shanxi	35.9767	111.5644	31.7	
307	Wanan, Jiangxian, Yuncheng, Shanxi	35.8559	111.5345	16.3	
308	Longxing, Xinjiang, Yuncheng, Shanxi	35.9621	111.2488	19.0	
309	Donghuan Rd., Xinjiang, Yuncheng, Shanxi	36.0025	111.2747	11.7	
310	Zhaokang, Xiangfen, Linfen, Shanxi	36.0367	111.4005	54.7	
311	Fengcheng, Houma, Linfen, Shanxi	36.1492	111.4065	39.3	
312	Shangma, Houma, Linfen, Shanxi	36.0017	111.6754	67.3	
313	Rougu, Yangling, Xianyang, Shaanxi	34.2705	108.0310	62.7	SS
314	NWUAF Campus, Xianyang, Shaanxi	34.4314	108.1092	136.7	
315	Wugong, Xianyang, Shaanxi	34.5566	108.2292	18.0	
316	Wugong, Xianyang, Shaanxi	34.5503	108.3135	51.0	
317	Qianxian, Xianyang, Shaanxi	34.6394	108.3477	23.0	
318	Qianxian, Xianyang, Shaanxi	34.7440	108.5506	8.3	
319	Jingyang, Xianyang, Shaanxi	35.0061	109.1365	2.7	

(continue)

No.	Sampling locality	Latitude	Longitude	CN	Ab
320	Zhouzhi, Zhouzhi, Xi'an, Shaanxi	34.1817	108.2094	57.0	SS
321	Xinglin, Fufeng, Baoji, Shaanxi	34.5839	108.5327	37.7	
322	Famen, Fufeng, Baoji, Shaanxi	34.7453	108.5165	80.3	
323	Yuechen, Meixian, Baoji, Shaanxi	34.4773	108.1135	32.7	
324	Shoushan, Baoji, Shaanxi	34.4301	108.2859	64.7	
325	Fengle, Liangzhou, Wuwei, Gansu	38.0459	102.6810	6.0	GS
326	Fengle, Liangzhou, Wuwei, Gansu	38.0554	102.6895	16.7	
327	Fengle, Liangzhou, Wuwei, Gansu	38.1166	102.6343	32.0	
328	Duoyuncun, Liangzhou, Wuwei, Gansu	38.1181	102.6342	10.3	
329	Wuhe, Liangzhou, Wuwei, Gansu	38.1119	102.7793	55.3	
330	Gaoba, Liangzhou, Wuwei, Gansu	38.0357	102.8482	31.7	
331	Liangzhou, Wuwei, Gansu	38.3574	103.1639	13.7	
332	Liangzhou, Wuwei, Gansu	38.2616	103.1918	3.7	
333	Liangzhou, Wuwei, Gansu	38.1541	103.3012	6.0	
334	Huangyang, Liangzhou, Wuwei, Gansu	38.0888	103.4552	24.3	
335	Shuangta, Gulang, Wuwei, Gansu	37.9975	103.4488	4.7	
336	Sishui, Gulang, Wuwei, Gansu	37.9976	103.4487	1.3	
337	Sishui, Gulang, Wuwei, Gansu	37.9939	103.5968	15.7	
338	Laoyoubao, Xining, Qinghai	38.0001	103.6158	2.3	QH
339	Xibao, Xining, Qinghai	36.8303	101.9732	7.7	
340	Shenzhong, Xining, Qinghai	36.8778	101.9405	2.7	
341	Bayan, Xining, Qinghai	37.2107	101.2972	4.3	
342	Bayan, Xining, Qinghai	37.2774	101.2247	11.0	
343	Huangyuan, Xining, Qinghai	37.3274	101.1732	3.3	
344	Tangchuan, Haidong, Qinghai	36.8283	101.2470	11.0	
345	Tangchuan, Haidong, Qinghai	36.6750	101.8878	3.7	
346	Xiegou, Datong, Xining, Qinghai	37.1223	102.4569	10.3	
347	Chengguan, Datong, Xining, Qinghai	37.0582	101.8761	8.0	
348	Lixian, Daxing, Beijing	39.5900	116.4079	25.0	BJ
349	Lixian, Daxing, Beijing	39.5980	116.4049	2.7	
350	Lixian, Daxing, Beijing	39.5941	116.4066	3.3	
351	Shigezhuang, Baodi, Tianjin	39.7260	117.2322	36.0	TJ
352	Shigezhuang, Baodi, Tianjin	39.7105	117.2429	32.0	
353	Sicundian, Wuqing, Tianjin	39.5121	116.9430	26.0	
354	Shizishan, Hongshan, Wuhan, Hubei	30.4759	114.3555	19.0	HB
355	Liupanshan, Jingyuan, Guyuan, Ningxia	35.6619	106.2940	24.0	NX
356	Shaogang, Qingtongxia, Wuzhong, Ningxia	38.1534	106.0649	7.0	
357	Hangjinhouqi, Bayan Nur, Inner Mongolia	40.7553	106.9924	5.0	NM
358	Wuchuanxian, Huhhot, Inner Mongolia	41.1499	111.3053	14.0	

(continue)

No.	Sampling locality	Latitude	Longitude	CN	Ab
359	Yongnian, Tianzhu, Wuwei, Gansu	37.1680	102.8380	81.9	TZ
360	Honggada, Tianzhu, Wuwei, Gansu	37.2190	102.7090	30.8	
361	Hongdaiganbai, Tianzhu, Wuwei, Gansu	37.2290	102.6950	11.0	
362	Chaxitan, Tianzhu, Wuwei, Gansu	37.1690	102.8380	17.7	
363	Anyuan, Tianzhu, Wuwei, Gansu	37.2050	102.8280	11.0	
364	Anyuan, Tianzhu, Wuwei, Gansu	37.2430	102.8440	9.4	
365	Shimen, Tianzhu, Wuwei, Gansu	36.9716	103.0113	1.0	
366	Dachaigou, Tianzhu, Wuwei, Gansu	37.0770	103.0060	17.5	
367	Gaza, Xiahe, Gannan, Gansu	35.3662	102.8720	7.2	
368	Lazetang, Xiahe, Gannan, Gansu	35.3588	102.8360	5.4	
369	Madang, Xiahe, Gannan, Gansu	35.3374	102.7894	2.0	
370	Wanggeertang, Xiahe, Gannan, Gansu	35.2242	102.7894	6.3	
371	Damai, Xiahe, Gannan, Gansu	35.2179	102.6935	16.5	
372	Kajiaman, Hezuo, Gannan, Gansu	35.0420	102.9159	2.7	
373	Nawu, Hezuo, Gannan, Gansu	35.0083	102.9435	14.0	

Table S2. Sampling locations and corresponding cysts recovered from the soil attached to trans-regional machinery harvesters.

No.	Sampling locality	Harvester transportation background	Coordination	Altitude	Weight of soil attached	Recovered cysts No.
1	Wulitou Village, Linying Chengguan Town, Luohe, Henan	From Zhumadian, Henan	33.79486, 113.92392	53 m	200 g	0
2	Shi Village, Linying Chengguan Town, Luohe, Henan	From Luohe, Wuyang, Henan	33.79708, 113.92639	54 m	200 g	0
3	Shi Village, Linying Chengguan Town, Luohe, Henan	From Luohe, Wuyang, Henan	33.80044, 113.92700	52 m	100 g	0
4	Shi Village, Linying Chengguan Town, Luohe, Henan	From Zhumadian, Henan	33.79914, 113.92992	52 m	100 g	0
5	Shi Village, Linying Chengguan Town, Luohe, Henan	From Zhumadian, Henan	33.79927, 113.93047	52 m	100 g	0
6	Xuchang, Changchun Zhang Town, Henan	From Sheqi, Nanyang	33.98325, 113.82794	63 m	100 g	0
7	Zhengzhou Agricultural Science Institute, Henan	Local harvester	34.76922, 113.46622	159 m	200 g	0
8	Baizhuang Town, Anyang, Henan	Local harvester	36.19420, 114.34173	59 m	100 g	1
9	Baizhuang Town, Anyang, Henan	Local harvester	36.20218, 114.36090	78 m	50 g	13
10	Jin Xiaozhuang, Baizhuang Town, Anyang, Henan	Local harvester	36.20263, 114.36085	78 m	100 g	0
11	Dongfanghong Village, Baizhuang, Anyang, Henan	Local harvester	36.18922, 114.35917	79 m	50 g	0
12	Taojiaying, Baizhuang Town, Anyang, Henan	From Nanyang and Xuchang	36.17603, 114.36302	73 m	200 g	1
13	Taojiaying, Baizhuang Town, Anyang, Henan	From Nanyang and Xuchang	36.17600, 114.36282	76 m	50 g	0
14	Gaozu, Baizhuang Town, Anyang, Henan	Local harvester	36.16823, 114.36043	72 m	100 g	3
15	Gaozu, Baizhuang Town, Anyang, Henan	Local harvester	36.16682, 114.35917	68 m	200 g	2
16	HouBaibi Town, Anyang, Henan Province	Local harvester	36.08923, 114.50917	94 m	100 g	4
17	HouBaibi Town, Anyang, Henan Province	Local harvester	36.09143, 114.51452	70 m	200 g	3
18	Gaopan Village, Baibi Town, Anyang, Henan	Local harvester	36.09563, 114.51317	66 m	100 g	13
19	Gaopan Village, Baibi Town, Anyang, Henan	Local harvester	36.09448, 114.51333	65 m	100 g	0
20	Chuwang Town, Anyang, Henan	From Changyuan, Hua to Wei	36.06878, 114.87225	49 m	100 g	0

21	Chuwang Town, Anyang, Henan	From Changyuan, Hua to Wei	36.06875, 114.87227	50 m	100 g	7
22	Chuwang Town, Anyang, Henan	From Changyuan, Hua to Wei	36.06897, 114.87230	51 m	100 g	19
23	Dongguan Village, Chengguan Town, Nanle, Henan	From Pingdingshan, Henan	36.06498, 115.23398	56 m	100 g	19
24	Dongguan Village, Chengguan Town, Nanle, Henan	From Pingdingshan, Henan	36.06598, 115.23518	54 m	100 g	0
25	Dongguan Village, Chengguan Town, Nanle, Henan	From Pingdingshan, Henan	36.06327, 115.23603	53 m	50 g	0
26	Nanjie Village, Zhanggutunp, Nanle, Henan	From Pingdingshan, Henan	36.04250, 115.29872	50 m	200 g	0
27	Nanjie Village, Zhanggutun, Nanle, Henan	From Xinxiang, Henan	36.04287, 115.29818	49 m	150 g	3
28	Han Zhang Town, West Han Village, Nanle, Henan	From Pingdingshan, Henan	36.05118, 115.32978	48 m	100 g	0
29	Henan Nanle, Hanzhang Town, Hangutuan	From Xinxiang, Henan	36.05127, 115.32872	49 m	100 g	3
30	Lijiatun Village, Gujinlou Township, Nanle, Henan	From Puyang, Henan	36.07587, 115.25035	48 m	250 g	15
31	Lijiatun Village, Gujinlou Township, Nanle, Henan	From Puyang, Henan	36.07595, 115.24980	48 m	100 g	0
32	Xifu Village, Xifu Township, Daming, Hebei	Local harvester, Henan	36.29625, 115.11517	67 m	100 g	0
33	Xifu Village, Xifu Township, Daming, Hebei	Local harvester, Henan	36.29672, 115.1149 7	66 m	100 g	1
34	Xifu Village, Xifu Township, Daming, Hebei	Local harvester, Henan	36.32367, 115.03942	54 m	200 g	64
35	Dayang Zhuang, Shakou Ji Township, Wei, Hebei	From Henan direction	36.32373, 115.03945	53 m	100 g	5
36	Dayang Zhuang, Shakou Ji Township, Wei, Hebei	From Henan direction	36.32573, 115.04022	52 m	200 g	113
37	Wangdonglibao, Heshan Town, Handan, Hebei	From Henan direction	36.49232, 114.61418	58 m	100 g	3
38	Jinchaao, Hesha Town, Handan, Hebei	From Henan direction	36.51073, 114.59927	56 m	100 g	0
39	Jinchaao, Hesha Town, Handan, Hebei	From Henan direction	36.50978, 114.59982	57 m	100 g	0
40	Yaozhuang, Shahe, Xingtai, Hebei	From Xinxiang, Henan	36.89260, 114.49845	63 m	100 g	5
41	Yaozhuang, Shahe, Xingtai, Hebei	From Xinxiang, Henan	36.89352, 114.49842	63 m	100 g	3
42	Yaozhuang, Shahe, Xingtai, Hebei	From Xinxiang, Henan	36.89402, 114.49835	64 m	100 g	19
43	Yaozhuang, Shahe, Xingtai, Hebei	From Xinxiang, Henan	36.89422, 114.49845	64 m	100 g	9

44	Anxia, Zhengding Town, Zhengding, Shijiazhuang, Hebei	From Handan, Hebei	38.18268, 114.57807	62 m	100 g	3
45	Anxia, Zhengding Town, Zhengding, Shijiazhuang, Hebei	Unknown	38.18372, 114.57635	64 m	100 g	0
46	Anxia, Zhengding Town, Zhengding, Shijiazhuang, Hebei	Unknown	38.18412, 114.57307	67 m	50 g	4
47	Yong'an, Zhengding Town, Zhengding, Shijiazhuang, Hebei	Unknown	38.18275, 114.56247	71 m	50 g	1

Table S3. The GenBank accession of obtained sequences and their corresponding haplotypes, host and origin. Multiple sequences were submitted for same haplotype as they are either in different lengths, or recognized as same haplotype in PopART. Sample codes are corresponding for the locations presented in table S1. Abbreviations: W=wheat, G=grass.

Accession	Haplotype	Length	Host	Regions	Sample code
ON357397	H8	791	W	HN, NM, SD	188-190,192 (2), 357, 255 (3)
ON357398	H7	791	W	BJ,TJ	348 (15), 351 (9)
ON357399	H6	791	W+G	GS, QH, TZ	325-330, 331 (3), 332 (3), 333, 334(3), 338 (2), 339, 340, 359, 360 (2)
ON357400	H7	791	W	SD	253 (5)
ON357401	H5	791	W	JSNT, JSTC	62. 63, 95-99, 120, 121
ON357402	H3	791	W	SS	313
ON357403	H1	791	W+G	AH, BJ, HB, HD, HN, JSNT, JSTC, NM, NX, SS, SD, SX, TJ, OYR, JSXZ	273-277, 278 (3), 279 (3), 280 (3), 281 (2), 282 (2), 350 (8), 354 (10), 283 (4), 284 (4),285 (2), 286 (2), 200 , 227, 230, 234 (3), 249 (2), 250 (2), 102 (3), 153 (2), 157 (2), 68 (6), 358 (8), 355 (10), 356 (7), 316 (5), 317 (4), 318 (4), 319 (2), 323 (2), 234 (2), 257-272, 302-310, 311 (3), 312 (7), 351 (4), 352 (3), 353 (3), 166-173, 11-13
ON357404	H1	791	W	HN	236, 239, 245
ON357405	H4	791	W	JSNT	105, 110, 118, 153 (2)
ON357406	H2	791	W	HD	288, 300 (2)
ON357407	H1	791	W	HD	287, 299
ON357408	H1	791	W	HD	290, 296, 297, 298
ON357409	H1	791	W	TJ	353
ON357410	H13	791	G	TZ	361-363, 364 (3)
ON357411	H13	791	G	TZ	366 (3), 367 (3), 368 (2), 369 (2)
ON357412	H17	791	W	JSTC	67
ON357413	H17	791	W	HD, JSNT, JSTC	287 (2), 288 (3), 293, 294, 111, 64 (3)
ON357414	H17	791	W	JSTC	65
ON357415	H19	791	W	QH	340, 343, 346 (2)
ON357416	H18	791	G	TZ	364, 370 (2), 371 (2)
ON357417	H20	791	G	TZ	365 (4)
ON357418	H20	791	G	TZ	370-373
ON357419	H11	791	W	NM, QH	358 (3), 342(2), 343-345, 347
ON357420	H5	445	W	JSTC	67 (3), 68
ON357421	H19	445	W	QH	346 (1)
ON357422	H1	445	W	HD	292, 64 (3)
ON357423	H11	445	W+G	GS, TZ, QH	329 (2), 330 (2), 333 (2), 360, 361, 338 (2), 339, 341 (2)
ON357424	H9	445	G	TZ	361 (6)
ON357425	H6	445	W	GS, QH	335, 346 (2)
ON357426	H4	445	W	JSTC	66 (3)
ON357427	H2	445	W	HD	290, 294 (3)

Table S4. The pairwise nucleotide difference between newly recovered mtCOI haplotypes.

	H9	H13	H7	H8	H6	H2	H3	H4	H5	H1	H19	H20	H18	H17	H11
H9		2	3	4	2	3	3	3	3	2	6	6	5	3	2
H13	2		9	10	8	13	13	13	13	12	12	12	11	11	10
H7	3	9		1	1	6	6	6	6	5	9	9	8	8	7
H8	4	10	1		2	7	7	7	7	6	10	10	9	9	8
H6	2	8	1	2		5	5	5	5	4	8	8	7	7	6
H2	3	13	6	7	5		2	2	2	1	13	13	12	12	10
H3	3	13	6	7	5	2		2	2	1	13	13	12	12	10
H4	3	13	6	7	5	2	2		2	1	13	13	12	12	10
H5	3	13	6	7	5	2	2	2		1	13	13	12	12	10
H1	2	12	5	6	4	1	1	1	1		12	12	11	11	9
H19	6	12	9	10	8	13	13	13	13	12		2	1	5	6
H20	6	12	9	10	8	13	13	13	13	12	2		1	5	6
H18	5	11	8	9	7	12	12	12	12	11	1	1		4	5
H17	3	11	8	9	7	12	12	12	12	11	5	5	4		5
H11	2	10	7	8	6	10	10	10	10	9	6	6	5	5	

Part 2: Morphometric

Table S5. Morphometrics of cyst and vulval cone of newly recovered Chinese cereal cyst nematode haplotypes. The morphometrics of eight haplotype H1 populations were compared, as they have different isolation origin. All measurements are in μm and in the form: mean ± s.d. (range).

Haplotype	H1	H1	H1	H1	H1	H1	H1	H1	H4
**Cyst (n)**	**15**	**15**	**15**	**15**	**15**	**15**	**15**	**17**	**17**
Length excl. neck	571 ±31<br>(511-623)	603 ±54<br>(520-685)	574 ±55<br>(454-644)	578 ±60<br>(480-684)	599 ±97<br>(412-765)	591 ±52<br>(479-658)	570 ±58<br>(468-661)	583 ±62<br>(463-667)	580 ±56<br>(473-651)
Width	352 ±50<br>(256-473)	361 ±26<br>(296-419)	356 ±49<br>(287-457)	375 ±40<br>(322-430)	379 ±63<br>(290-514)	381 ±37<br>(308-447)	352 ±37<br>(290-411)	437 ±43<br>(333-520)	411 ±56<br>(330-517)
Length/Width	1.6 ±0.2<br>(1.3-2.3)	1.7 ±0.2<br>(1.5-2)	1.6 ±0.14<br>(1.4-1.9)	1.6 ±0.2<br>(1.3-2)	1.6 ±0.2<br>(1.4-1.9)	1.56 ±0.1<br>(1.3-1.8)	1.6 ±0.2<br>(1.4-1.9)	1.3 ±0.2<br>(1.1-1.6)	1.4 ±0.2<br>(1.2-1.8)
**Vulval cone (n)**	**10**	**12**	**11**	**12**	**9**	**10**	**8**	**14**	**12**
Fenestral length	39 ±2.5<br>(34-44)	40 ±3.2<br>(35-45)	41 ±3.1<br>(37-47)	43 ±3.8<br>(33-48)	45 ±2.8<br>(40-50)	42 ±2.9<br>(38-46)	40 ±1.6<br>(38-42)	44 ±3.9<br>(37-50)	39 ±5.4<br>(31-48)
Mean semifenestral width	21 ±1.6<br>(17-22)	20 ±1.2<br>(17-21)	19 ±1.4<br>(17-22)	22 ±1.7<br>(20-25)	21 ±0.9<br>(20-23)	20 ±1.4<br>(18-22)	22 ±1.3<br>(20-24)	22 ±2.7<br>(18-27)	21 ±3.9<br>(14-27)
Vulval bridge width	7.0 ±0.8<br>(5.6-8.0)	6.3 ±1.01<br>(4.5-7.8)	6.6 ±0.7<br>(6-7.7)	8 ±0.7<br>(6.7-9.3)	7.1 ±1.1<br>(5.-9.1)	7.5 ±1.0<br>(6.7-9.5)	6.93 ±0.6<br>(5.9-7.6)	9.0 ±1.6<br>(6.2-11.3)	8.4 ±1.9<br>(5.3-10.9)
Vulval slit length	10 ±1.3<br>(7.4-12)	9.3 ±1.2<br>(7.6-11.5)	10 ±1.4<br>(7.1-12)	11 ±1.4<br>(8.9-13)	10.2 ±1.3<br>(7.7-11.8)	9.4 ±1.1<br>(7.8-11)	9.6 ±1.02<br>(8.2-11)	8.9 ±1.3<br>(6.4-11)	8.6 ±1.6<br>(5.3-11.1)
Vulval-anus distance	48 ±6.0<br>(39-56)	46 ±4<br>(38-52)	45 ±3.8<br>(40-54)	47. ±4.6<br>(39-53)	46 ±1.9<br>(43-49)	44 ±4.4<br>(37-51)	46 ±4.3<br>(38-52)	47 ±6.1<br>(36-55)	46 ±4.9<br>(38-53)

(continue)

Haplotype	H5	H13	H9	H20	*H. avenae* Fangshan, China *	*H. avenae* Ha-hoola, Israel**	*H. avenae* Cukurova Turkey**	*H. avenae* Taaken Germany**	*H. pratensis* Otterndorf, Germany*
**Cyst (n)**	**17**	**4**	**4**	**4**	**9**	**15**	**25**	**30**	**5**
Length excl.neck	593 ±47 (511-687)	671 ±35 (618-691)	594 ±24 (575-627)	533 ±95 (425-607)	597 ±25 (504-696)	835 ±34 (552-1056)	845 ±22 (672-1056)	709 ±19.3 (488-896)	672 ±67 (408-768)
Width	427 ±52 (332-554)	510 ±65 (456-580)	445 ±80 (356-550)	403 ±90 (335-368)	437 ±13 (384-504)	589 ±23 (408-720)	601 ±16 (432-744)	490 ±13.7 (360-584)	523 ±62 (288-624)
Length/Width	1.4 ±0.1 (1.2-1.8)	1.3 ±0.14 (1.2-1.6)	1.4 ±0.19 (1.1-1.6)	1.3 ±0.18 (1.2-1.5)	1.4 ±0.05 (1.2-1.5)	1.4 ±0.03 (1.1-1.6)	1.4 ±0.03 (1.2-1.7)	1.5 ±0.02 (1.3-1.7)	1.3 ±0.04 (1.2-1.4)
**Vulval cone (n)**	**11**	**4**	**4**	**4**	**10**	**12**	**5**	**20**	**5**
Fenestral length	43 ±5.1 (36-52)	46 ±2.8 (43-49)	47 ±0.49 (46-47)	39 ±7.6 (31-45)	46 ±0.8 (43-50)	53 ±1.1 (47-58)	55 ±1.7 (51-59)	47.8 ±0.7 (42.5-52.5)	45 ±0.9 (43-47)
Semifenestral width	21 ±3.9 (14-26)	25 ±4.1 (21-28)	27 ±2.6 (24-30)	24 ±5.3 (20-30)	23 ±1.3 (19-31)	25 ±0.7 (19-28)	21 ±1.0 (20-23)	24.8 ±0.6 (20-30)	22 ±0.9 (19-23)
Vulval bridge width	9.2 ±1.6 (6.5-12.2)	7.0 ±0.79 (6.0-7.6)	6.4 ±0.7 (5.4-6.9)	6.4 ±0.76 (5.3-7.1)	10 ±0.9 (6.6-16)	12 ±0.7 (7.8-16)	12 ±1.1 (7.8-14)	7.3 ±0.2 (6.3-7.5)	8.5 ±0.4 (7.8-9.7)
Vulval slit length	9.6 ±1.5 (7.4-11.5)	10.0 ±1.6 (8.3-12)	10.4 ±0.53 (5.4-6.9)	10 ±1.2 (8.5-11)	8.6 ±0.4 (6.6-12)	8.5 ±0.2 (7.8-9.7)	10 ±1.0 (7.8-12)	10.1 ±0.3 (8.0-12.5)	7.6 ±0.7 (5.8-9.7)
Vulva to anus distance	49 ±5.9 (40-59)	49 ±6.6 (39-58)	46 ±3.5 (42-50)	48 ±5.6 (44-52)	57 ±1.5 (50-62)	49 ±2.5 (43-62)	55.70	-	49 ±3.4 (43-54)

Note: n = Number of specimens;

* Measurements according to Subbotin *et al*. (2015).

** Measurements according to Subbotin *et al*. (2003).

Table S6. Morphometrics for second-stage juveniles of newly recovered Chinese CCN haplotypes. The morphometrics of eight haplotype H1 populations were compared, as they have different isolation origin. All measurements are in μm and in the form: mean ± s.d. (range).

Haplotype	H1	H1	H1	H1	H1	H1	H1	H1	H4	H5
**n**	**10**	**10**	**10**	**10**	**10**	**10**	**10**	**12**	**12**	**12**
L	538 ±21<br>(502-566)	558 ±17<br>(536-593)	532 ±22<br>(491-560)	535 ±30<br>(501-584)	524 ±21<br>(501-563)	519 ±18<br>(486-543)	527 ±17<br>(493-554)	560 ±10<br>(542-575)	563 ±16<br>(534-583)	551 ±23<br>(521-581)
a	23 ±0.01<br>(0.2-0.25)	20 ±1.2<br>(18-22)	23 ±1.6<br>(20-25)	21.9 ±1.8<br>(20-24)	24 ±1.6<br>(21-27)	23 ±1.7<br>(20-25)	21 ±0.01<br>(0.2-0.24)	24 ±1.6<br>(22-26)	25 ±1.0<br>(24-27)	25 ±0.9<br>(23-26)
b	5.9 ±0.3<br>(5.6-6.5)	5.8 ±0.3<br>(5.3-6.5)	5.7 ±0.4<br>(5-6)	5.7 ±0.2<br>(5.4-6)	5 ±0.4<br>(4.8-6.4)	5.7 ±0.3<br>(5.4-6.2)	5.5 ±0.2<br>(5-5.9)	5.4 ±0.3<br>(5-5.8)	5.8 ±0.3<br>(5.3-6.4)	5.9 ±0.3<br>(5.4-6.4)
c	8.6 ±0.6<br>(7.9-9.7)	8.6 ±0.5<br>(7.7-9.3)	8.2 ±0.2<br>(7.8-8.6)	8.8 ±0.23<br>(8.4-9.2)	8.6 ±0.5<br>(7.8-9.2)	8.4 ±0.4<br>(7.8-9.0)	8.7 ±0.5<br>(7.8-9.6)	9.3 ±0.7<br>(8.5-11.1)	8.2 ±0.5<br>(7.7-9.1)	9.0 ±0.6<br>(8.4-10.3)
Stylet length	24 ±0.6<br>(23-25)	25 ±0.7<br>(24-25)	24 ±0.7<br>(23-25)	24 ±0.9<br>(23-26)	23 ±0.7<br>(22-25)	24 ±0.6<br>(23-25)	24 ±0.9<br>(23-26)	25 ±0.7<br>(24-26)	25 ±0.6<br>(24-26)	25 ±0.9<br>(23-26)
Lip region width	9.8 ±0.3<br>(9.3-10)	9.9 ±0.5<br>(9.3-10)	9.6 ±0.3<br>(9.2-10)	9.8 ±0.5<br>(9.1-10)	9.7 ±0.4<br>(9-10)	9.3 ±0.3<br>(9-9.9)	9.6 ±0.4<br>(9.1-10)	9 ±0.4<br>(8.2-9.6)	9.5 ±0.3<br>(9.1-9.9)	9.2 ±0.5<br>(8.4-10.1)
Lip region height	3.5 ±0.2<br>(3.3-3.9)	3.6 ±0.2<br>(3.3-3.9)	3.4 ±0.2<br>(3-3.7)	3.3 ±0.2<br>(3.1-3.7)	3.4 ±0.3<br>(3.1-3.8)	3.4 ±0.2<br>(3.1-3.8)	3.6 ±0.2<br>(3.3-3.8)	3.8 ±0.3<br>(3.4-4.1)	4.1 ±0.2<br>(3.8-4.4)	4.0 ±0.4<br>(3.6-4.8)
DGO	5.4 ±0.2<br>(5.1-5.9)	5.5 ±0.2<br>(5.1-5.8)	5.5 ±0.2<br>(5.28-5.84)	5.3 ±0.2<br>(5-5.7)	5.2 ±0.2<br>(5.0-5.6)	5.3 ±0.3<br>(4.8-5.6)	5.5 ±0.2<br>(5.2-5.8)	5.7 ±0.4<br>(5-6.2)	6.2 ±0.6<br>(5.1-7.1)	6.6 ±0.8<br>(5.3-7.9)
Anterior end to excretory pore	104 ±6.5<br>(91-113)	112 ±4.12<br>(104-119)	105 ±3.3<br>(101-112)	110 ±4.8<br>(102-116)	106 ±3.65<br>(98-110)	103 ±2.7<br>(98-108)	109 ±3.3<br>(102-115)	113 ±4.2<br>(105-121)	110 ±2.7<br>(105-114)	105 ±3.6<br>(101-111)
BWA	15 ±0.8<br>(14-16)	18 ±1.4<br>(15-20)	16 ±0.9<br>(14-18)	16 ±1.4<br>(15.3-19)	16 ±0.4<br>(14-16)	16 ±0.6<br>(15-17)	17 ±1.7<br>(15-19)	16 ±0.9<br>(14-17)	16 ±0.6<br>(15-17)	15 ±0.7<br>(14-16)
Tail length	63 ±4.8<br>(52-68)	65 ±4.4<br>(58-72)	65 ±3.5<br>(60-70)	61 ±4.3<br>(57-67)	61 ±3.5<br>(56-67)	62 ±3.1<br>(56-66)	61 ±4.1<br>(51-67)	60 ±4.3<br>(51-66)	69 ±4.8<br>(61-76)	61 ±3.3<br>(55-65)
Hyaline tail length	39 ±3.7<br>(35-47)	40 ±3.8<br>(33-46)	41 ±4.5<br>(35.4-48)	39 ±3.3<br>(34-44)	39 ±2<br>(35-42)	39 ±2.7<br>(36-43)	37 ±2.5<br>(32-40)	38 ±3.7<br>(33-45)	44 ±3.1<br>(41-49)	42 ±2.8<br>(40-48)
Tail length/BWA	4.1 ±0.2<br>(3.8-4.4)	3.6 ±0.4<br>(3.1-4.2)	4.2 ±0.3<br>(3.4-4.5)	3.7 ±0.12<br>(3.5-3.9)	3.9 ±0.2<br>(3.6-4.3)	3.9 ±0.2<br>(3.6-4.3)	3.5 ±0.4<br>(3.2-4.2)	3.9 ±0.3<br>(3.3-4.4)	4.3 ±0.3<br>(3.9-4.9)	4.1 ±0.3<br>(3.7-4.6)

Note: n = Number of specimens; L = Total body length; a = Body length/body width; b = Body length/distance from anterior end to junction of oesophagus and intestine; c = Body length/tail length; BWA = Body width at anus; DGO = Dorsal pharyngeal gland opening from stylet konbs.

Part 3:

Analysis of genetic structure for Chinese *Heterodera avenae* populations based on SSR microsatellite markers

Material and methods

DNA extraction

The cysts were recovered from wheat root samples collected at different locations (Table S1). Prior to DNA extraction, the identity of recovered Chinese *Heterodera avenae* population (CHA) were confirmed both by morphology and molecular barcoding using COI gene. The cysts were incubated at 4 °C for 6 weeks then at 16 °C to stimulate the hatching of pre-parasitic second-stage juveniles (J2s). For each population, J2s from 32 individual cysts were extracted separately following the protocol described by Adam et al. (2007), resulting a total of 416 individual cysts from 371 samples were used further analyses (Table S1).

SSR amplification and genotyping

A total of 9 pair primers were used for SSR amplification (Table S7), and subsequently divided into three groups according fragment size, i.e. (1) CS-19, CS-20, CS-31; (2) CS-60, CS-137, CS-170; (3) CS-179, CS-8384, CS-174. PCR was performed following the method detailed in Wang et al. (2018). Briefly, a 20 µL reaction mixture was used containing three primers and 2 µL of the template DNA of each cyst, 0.2mMdNTP, 1 × PCR buffer and 0.2 µL TaKaRa Ex Taq DNA Polymerase (Takara Bio, Dalian, China). PCR products were analyzed using an ABI 3730 sequencer (Applied Biosystems, Carlsbad, CA, USA) according to the manufacturer's instructions. Allele sizes were determined using GENEMAPPER version 4.0 (Applied Biosystems, Carlsbad, CA, USA), using LIZ-500 (−250) as the size standards.

Table S7 Primer sequence and features of nine microsatellite loci used in this study

Locus	Motif	Primer sequence (5'-3')	T_a (°C)	Size range (bp)
CS-19	$(AGG)_5$	F：ATGAGCAGCAACAACAGCAG	56	147－151
		R：ACTTCTTCATCGTGTTCCGTATG		
CS-20	$(TG)_{10}$	F：ATTCGAATACTTCTGCTCTTCCC	56	128－155
		R：GAATGGAAGGCGTAACTAAGGTT		
CS-31	$(CTG)_6$	F：ACAGTATGGCTATCAGCAAAAGG	56	164－168
		R：TTCTGCTCCAAATTGTCTTCTTC		

CS-60	$(TTC)_5$	F：AAACCCGAATCAAAAGAAAGAAG	56	139－164
		R：AAAATGGGTTGGAGAAGAAGAAG		
CS-137	$(GTT)_5$	F：ACACTCTCTTCCGACTCCTCC	56	173－179
		R：GTTCATCGACAGCAATCACAAT		
CS-170	$(TGT)_7$	F：CTGTTGCCGATGTTGTTGTC	56	151－154
		R：CAACTTCCTGATCAGCAGTCATA		
CS-174	$(GAAT)_6$	F：AAAAGTTTGTCGACCATTGAAAA	56	160－174
		R：ATCTCCATTTTGGTCATCACATC		
CS-179	$(AG)_{10}$	F：CGGAGTACGGTAGTGAAAGATTG	56	155－169
		R：GTCATTCAATCCTCCTCCTTCTT		
CS-8384	$(CT)_{10}$	F：GAAGACCATCAAATGGACTCAAC	56	163－173
		R：AGAGACAAATGGACGACAACACT		

Data analyses

The FSTAT 2.9.3 program (Goudet, 1995) was used to calculate the fixation index (F_{ST}) and the individual inbreeding coefficient (F_{is}) among and within the population. The MSAnalyzer 4.05 (Dieringer & Schlötterer, 2003) was used to calculate the genetic distance between J2 individuals based on shared alleles, and GenAlex 6.5 (Peakall & Souse, 2006) was used for PCoA analysis using the genetic distance matrix. STRUCTURE v2.3.4 was used to analyze the population structure based on the Bayesian clustering method (Pritchard et al., 2000), and the mixed ancestry model and the allele frequency association model were used to carry out 10^6 repeated Markov Monte Carlo Chain (MCMC) search, and discard the first 10^5 clustering results to determine the optimal number of clusters (K).The K value is set from1 to 13, and each K value is run 20 times to calculate the LnP(D) corresponding to each K value, and then calculate the ΔK value through LnP(D) to obtain the difference between the K value and ΔK (Evanno et al., 2005). The 20 replicates were aligned and integrated using CLUMPP v1.1.2 software (Jakobsson & Rosenberg, 2007), and finally plotted using DISTRUCT (Rosenberg, 2004). The ARLEQUIN v3.5 (Excoffier et al., 2010) was used to calculate the Analysis of Molecular Variance (AMOVA), and the three types were compared: variation between groups, between populations but within a group, and among individuals but within a population. Neighbor-joining phylogenetic tree was constructed using POPULATION v1.2.30 (http://bioinformatics.org/~tryphon/populations) based on Cavalli-Sforza and Edwards genetic distances (Cavalli-Sforza & Edwards, 1967). The

Wilcoxon signed-rank test method in BOTTLENECK v1.2.02 (Cornuet & Luikart, 1996) was used to test whether each population had experienced bottleneck effect, based on infinite allele model (IAM) and two-phase model (TPM) and stepwise mutation model (SMM), and three models were tested separately (Cristescu et al., 2010). In order to clarify whether geographic isolation aggravates the graminearum cyst nematode Genetic differentiation of populations. The Mantel test implied R package "ade4" was used to test the fixation index F_{ST} among populations based on microsatellite data and the average geographic distance of the sampling area.

Results

Genetic diversity of the CHA population

In general, we recovered a low genetic diversity among studied population (Table S8). The result showed that the 13 CHA populations have the allelic richness ranging from 1.889 to 3.111, with an average of 2.453. The observed heterozygosity was ranging from 0.163 to 0.344 with an average of 0.253 while the expected heterozygosity was between 0.180 and 0.414 with an average was 0.276. Testing for Hardy-Weinberg equilibrium found that 8 out of 13 populations deviate from the equilibrium and the main reason for the deviation is the loss of heterozygotes. The inbreeding coefficient (F_{is}) ranged from -0.115 to 0.220, and the rest of the populations were deviate significantly from equilibrium (P < 0.05) except for the Gansu population (GS), Qinghai population (QH), Hebei population (HH), Shandong population (SD) and Hubei population (HB).

Table S8 Statistics of genetic diversity for 13 CHA populations based on nine microsatellite markers

Population	*N*	*N*a	*N*e	*H*o	*H*e	u*H*e	HWE	*Fis*
TJ	32	3.111	1.900	0.344	0.414	0.421	***	0.185
BJ	32	2.677	1.827	0.319	0.386	0.393	***	0.187
GS	32	1.889	1.451	0.264	0.239	0.243	ns	-0.088
QH	32	2.778	1.242	0.192	0.180	0.183	ns	-0.044
SS	32	2.333	1.164	0.281	0.339	0.345	*	0.187
NX	32	2.000	1.368	0.163	0.205	0.208	**	0.219
SX	32	2.333	1.449	0.205	0.228	0.231	***	0.115
HH	32	2.111	1.426	0.260	0.230	0.234	ns	-0.115
SD	32	2.333	1.442	0.243	0.238	0.242	ns	-0.004
AH	32	2.111	1.469	0.188	0.236	0.240	**	0.220
HN	32	3.000	1.743	0.299	0.342	0.347	***	0.143
HB	32	2.333	1.480	0.267	0.257	0.261	ns	-0.023
JS	32	2.889	1.507	0.271	0.299	0.304	**	0.110
Mean		2.453	1.534	0.253	0.276	0.281		

Sample size (*N*), average number of alleles per locus (*N*a), the effective number of alleles (*N*e), the observed heterozygosity (*H*o), the expected heterozygosity (*H*e), unbiased expected heterozygosity (u*H*e), deviation from HWE for heterozygosity deficit and estimator of the fixation index (*F*is); ns, not significant, * $P < 0.05$, ** $P < 0.01$, *** $P < 0.001$

Population structure of CHA

Genetic structure of studied population suggested a moderate to high F_{ST} value, with the average fixation index as 0.162, and the 95% confidence interval is between 0.094 and 0.224 (Table S9). The smallest fixation index (F_{ST} = 0.004) was found between Beijing (BJ) and Tianjin (TJ) while the largest (F_{ST} = 0.435) was between Qinghai (QH) and Ningxia (NX) population. Mantel test was performed using F_{ST} as the genetic distance against their geographic distance. The results suggested that there was a significant positive correlation between genetic distance and geographic distance (r = 0.284, P = 0.038), and the CHA population followed the isolation-by-distance pattern (IBD) pattern (Fig. S2).

Table S9 Pairwise comparison of F_{ST} values between different CHA populations

Population	TJ	BJ	GS	QH	SS	NX	SX	HD	SD	AH	HN	HB
BJ	0.004											
GS	0.122*	0.117*										
QH	0.129*	0.153*	0.117*									
SS	0.109*	0.101*	0.172*	0.239*								
NX	0.234*	0.209*	0.350*	0.435*	0.075*							
SX	0.252*	0.249*	0.381*	0.395*	0.132*	0.182*						
HH	0.202*	0.191*	0.331*	0.364*	0.130*	0.142*	0.084*					
SD	0.120*	0.120*	0.247*	0.239*	0.110*	0.140*	0.156*	0.050*				
AH	0.159*	0.185*	0.343*	0.346*	0.150*	0.209*	0.226*	0.182*	0.129*			
HN	0.095*	0.068*	0.170*	0.261*	0.052*	0.086*	0.166*	0.085*	0.073*	0.114*		
HB	0.134*	0.124*	0.262*	0.322*	0.063*	0.055*	0.153*	0.070*	0.045*	0.088*	0.024*	
JS	0.060*	0.061*	0.175*	0.190*	0.042*	0.119*	0.142*	0.125*	0.057*	0.079*	0.045*	0.039*

* $P < 0.05$

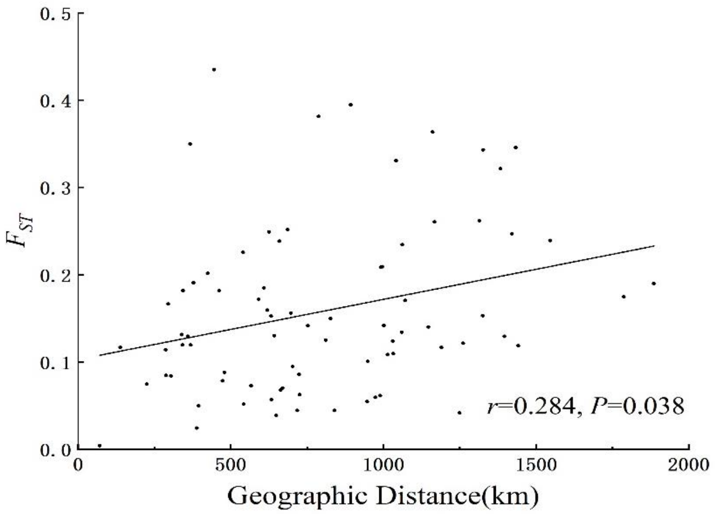

Fig. S2 Mantel test of correlation between geographic distance and genetic distance of CHA populations

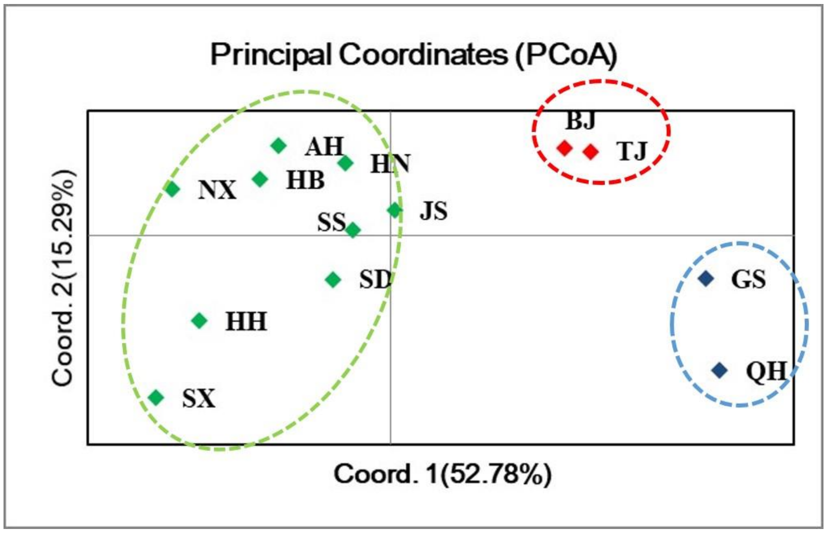

Fig. S3 Scatterplot of the principal coordinate (PcoA) of CHA populations based on SSR data

The PcoA (Fig. S3) showed that the CHA populations were clustered into three groups, *i.e.*: Beijing-Tianjin region, central region and northwest region. With the coordinate 1 explains 52.78% and coordinate 2 explains 15.29% of variance, two axes explain a total of 68.07% variance. We further constructed the NJ phylogenetic tree based on the Cavalli-Sforza & Edwards genetic distance and resulted topology was generally consistent with the clustering in principal coordinate analysis (Fig. S4).

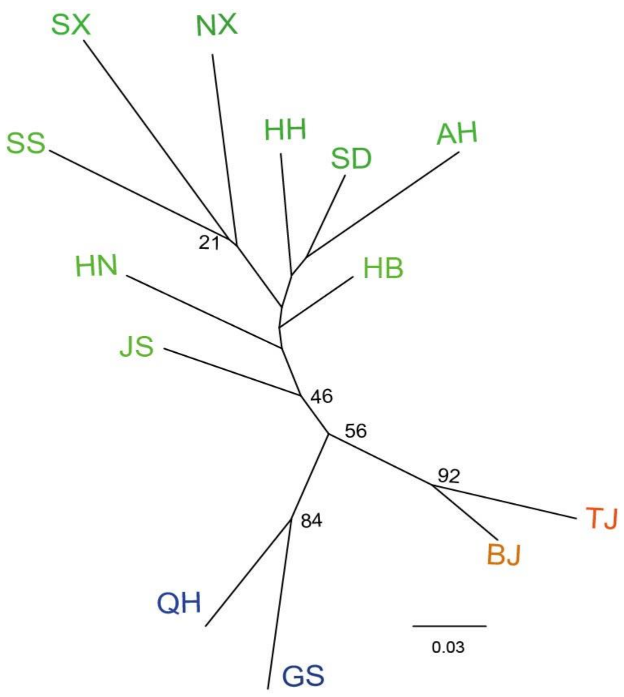

Fig. S4 Unrooted NJ tree based on SSR data from CHA populations

In Bayesian cluster analysis based on STRUCTURE, the highest △K value was achieved when K = 2 (Figure 5a), but △K remain relatively high when K = 3. Only after the LnP(K) value greater than 3 both △K and LnP(K) tends to be stable (Fig. 5a,4b).

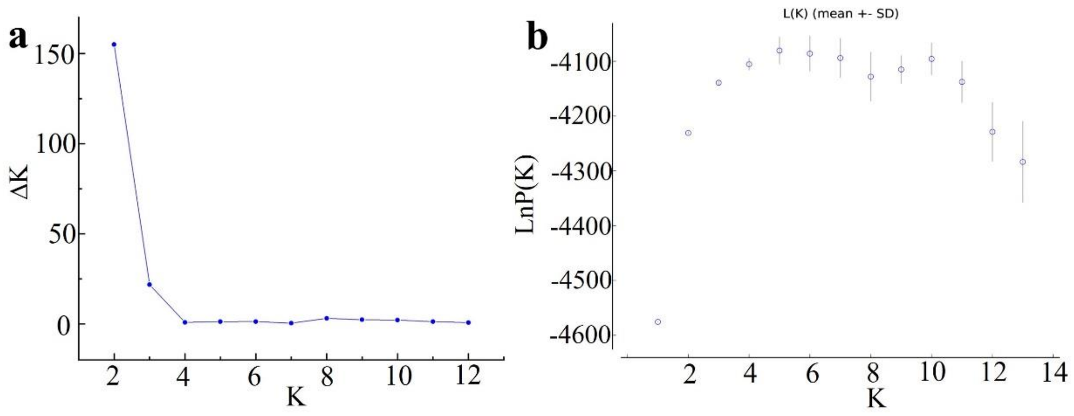

Fig. S5 Clusters number (K) based on Bayesian inference. The posterior probability of the SSR data were used to estimate K value with each K being 20 replicates (a) and the △K distribution (b)

When K = 2, the studied populations can be clustered into two groups, namely the Beijing-Tianjin-Northwest (green, Group I) and the central region (red, Group II), and the gene mixing is more obvious. When K = 3, the populations were clustered into three groups, namely the

Beijing-Tianjin region (Clade I), northwest region (Clade II) and central region (Clade III). All groups within Clade II and Clade II groups had mixed genotypes (Fig. S6).

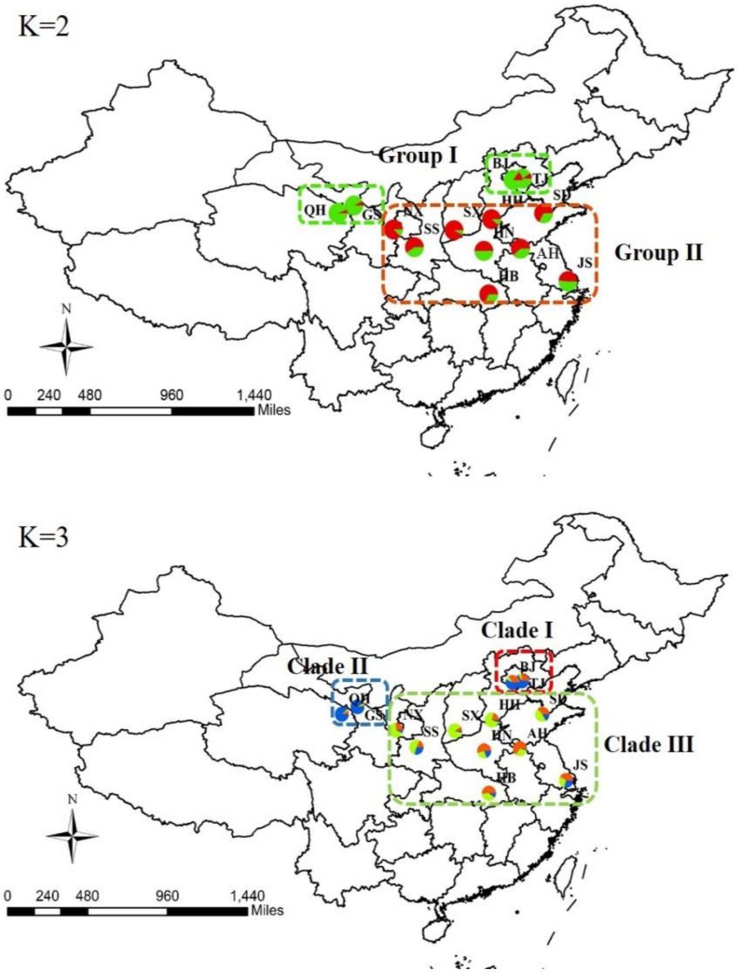

Fig. S6 The distribution of genotype in different clustering K value

The analysis of molecular variation model

The results of AMOVA (Table S10) showed that 83.77% of the variation came within the population (intra-population) in the case of no grouping. When the population was clustered into two groups (K = 2), the intra-population variation rate accounted for 78.03%, the inter-groups variation rate was 12.72%, and the inter-populations variation rate was 9.25%. When the population was clustered into 3 groups (K = 3), the population variation rate was similar to that of the two groups, the intra-population variation rate accounted for 78.09%, and the inter-group variation rate was 13.91%, and the variation rate between populations was 7.99%.

Table S10 AMOVA analysis of CHA populations

Source of variation	*df*	Sum of squares	Variance components	Percentage of variation (%)	F_{ST}
Overall					
Inter-population	12	203.215	0.245 Va	16.23	0.162*
Intra-population	819	1035.031	1.264 Vb	83.77	
Two groups (k=2)					
Inter-groups	1	83.891	0.206 Va	12.72	0.220*
Inter-population	11	119.324	0.150 Vb	9.25	0.110*
Intra-population	819	1035.031	1.264 Vc	78.03	0.127*
Three groups (k=3)					
Inter-groups	2	107.778	0.225 Va	13.91	0.139*
Inter-population	10	95.438	0.129 Vb	7.99	0.093*
Intra-population	819	1035.031	1.264 Vc	78.09	0.219*

* P<0.001.

Detection of possible bottleneck effect

The BOTTLENECK software was used to detect the bottleneck effect was calculated based on the three models of IAM, TPM and SMM. The data are shown in Table S11.

Table S11 Bottleneck effect of CHA populations based on Wilcoxon test

Population	IAM	TPM	SMM
AH	0.406	0.531	0.656
BJ	0.082	0.282	0.410
GS	0.039*	0.219	0.344
HB	0.422	0.680	0.963
HH	0.234	0.531	0.813
HN	0.422	0.629	0.844
JS	0.752	0.898	0.982
NX	0.344	0.719	0.781
QH	0.998	0.999	0.999
SS	0.027*	0.125	0.371
SD	0.680	0.844	0.963
SX	0.727	0.844	0.981
TJ	0.102	0.285	0.590
Beijing-Tianjin	0.203	0.734	1.000

Northwest region	0.426	0.203	0.020*
Central region	0.570	0.203	0.090
Overall	0.910	0.359	0.057

* $P < 0.05$

In general, no significant bottleneck effect was found in across populations according to TPM and SMM model. However, signals were detected under the IAM model suggesting the GS and SS populations may recently experience bottleneck effects. Under the SMM model and three clustering groups, only the northwest region has a certain bottleneck effect, while neither the Beijing-Tianjin region nor the central region has experienced the bottleneck effect.

References

Adam M A M，Phillips M S，Blok V C．Molecular diagnostic key for identification of single juveniles of seven common and economically important species of root-knot nematode (*Meloidogyne* spp)．Plant Pathology, 2007, 56: 190–197．

Cavalli-Sforza L L，Edwards A W F．Phylogenetic analysis-models and estimation procedures. American Journal of Human Genetics, 1967, 21: 550-570.

Cornuet J M，Luikart G．Description and power analysis of two tests for detecting recent population bottlenecks from allele frequency data. Genetics, 1996, 144: 2001-2014.

Dieringer, D. and Schlötterer C. Microsatellite analyzer (MSA): a platform independent analysis tool for large microsatellite data sets. Molecular Ecology Notes, 2003, 3:167-169.

Evanno G, Regnaut S, Goudet J. Detecting the number of clusters of individuals using the software STRUCTURE: a simulation study. Molecular Ecology, 2005, 14: 2611-2620.

Excoffier L，Lischer H E L．Arlequin suite ver 3.5: a new series of programs to perform population genetics analyses under Linux and Windows．Molecular Ecology Resources, 2010, 10: 564-567.

Goudet J. FSTAT (Version 1.2): A computer program to calculate F-statistics．Journal of Heredity, 1995, 86: 485-486.

Jakobsson M，Rosenberg N A．CLUMPP: a cluster matching and permutation program for dealing with label switching and multimodality in analysis of population structure. Bioinformatics, 2007, 23: 1801-1806.

Peakall R, Smouse P E. GENALEX 6: genetic analysis in Excel．Population genetic software for teaching and research．Molecular Ecology Resources, 2006, 6: 288-295.

Pritchard JK，Stephens M，Donnelly P．Inference of population structure using multilocus genotype data．Genetics, 2000, 155: 945–959.

Rosenberg, N. A. DISTRUCT: a program for the graphical display of population structure. Molecular Ecology Notes, 2004, 4:137-138.

Wang, X., Ma, J., Liu, H., Liu, R., and Li, H. Development and characterization of EST-derived SSR markers in the cereal cyst nematode Heterodera avenae. European Journal of Plant Pathology, 2018, 150, 105-113.

Part 4: Material and method

1. *Morphological analyses*

Cysts were extracted from approximately 500 g of soil samples using standard flotation and sieving technique. To examine the floating cysts in water, a 100 mesh sieve was placed under water for 2-4 h. For morphological analyses, second-stage juveniles (J2) were extracted from soil using Baermann tray, killed and fixed in hot TAF and processed to glycerin dehydration (De Grisse, 1969). The J2s were mounted in glycerin and vulval cones were mounted in gelatine. The permanent slides were examined, measured and photographed with Olympus BX51 microscopy equipped with Olympus DP72 camera.

2. *PCR amplification and sequencing*

Two primer pairs were used for COI gene amplification. A shorter primer pairs JB3 (5'-TTT TTT GGG CAT CCT GAG GTT TAT-3') and JB4.5 (5'-TAA AGA AAG AAC ATA ATG AAA ATG-3') (Bowles et al., 1992), and a newly designed longer primer pairs COI442F (5'-CAT TTA GCA GGA ATT AGT TC-3') and COI1326R (5'-CAC TAT AAT CTA AAT ATT TAC G-3'). Reaction mixture is 25 µl in volume containing the following: 2.5 µl 10× buffer, 1.5 µl 50 mM MgCl2, 2.0 µl 2.5 mM dNTP, 1 U Taq DNA polymerase (TaKaRa, China), 1 µl 10 µM of each primer, 2-4 µl genomic DNA (50 ng/µl), and double-distilled water was added to make a final volume of 25 µl. PCR reaction was conducted at 95°C for 3 min, followed by 35 cycles of 30 s at 95 ℃, 30 s at 45 ℃, 30 s at 72 ℃, and finally at 72 ℃ for 10 min. The amplified products were examined by agarose gel electrophoresis and subsequently sent to TsingKe Biotechnology (Nanjing, China) for sequencing.
